# DEG2MOL: Conditional Latent Flow Matching for Transcriptome-Guided *de novo* Drug Design

**DOI:** 10.1021/acsomega.6c07302

**Published:** 2026-09-28

**Authors:** Songhyeon Kim, Wootaek Lim, Hawon Lee, Hyunsu Bong, Jitae Kim, Minji Jeon

**Affiliations:** † Department of Biomedical Sciences, 36899 Korea University College of Medicine, 73 Goryeodae-ro, Seoul 02841, Republic of Korea; ‡ Department of Biomedical Informatics, Korea University College of Medicine, 161 Jeongreung-ro, Seoul 02708, Republic of Korea

## Abstract

Transcriptomic profiles capture cellular responses to perturbations, offering a principled foundation for biologically grounded *de novo* drug design. However, existing transcriptome-guided generative approaches remain limited in biological specificity, transcriptomic coverage, and evaluation across diverse perturbation contexts. Here, we propose DEG2MOL, a biologically grounded conditioning framework that encodes differentially expressed gene profiles through a Gene Ontology (GO)-informed encoder and maps this representation into the latent space of a frozen, pre-trained scaffold-aware VAE via conditional latent flow matching. When benchmarked against five baseline models, DEG2MOL ranked first by rank-sum aggregation of six metrics in both the random- and scaffold-split evaluations. For two representative compound conditions, GO-informed encoder activations were accompanied by enrichment of the corresponding target-engaging pharmacophores among the generated molecules. In docking case studies of two compound–target pairs, generated molecules showed favorable predicted docking scores and reference-associated contacts. DEG2MOL also generated molecules from shRNA knockdown, CRISPR knockout, and Perturb-seq profiles without additional training, indicating transfer across the perturbation types and profiling platforms evaluated. At the transcriptional level, an *in silico* gene-expression analysis showed that the predicted signatures of generated molecules were more similar to those of target-matched inhibitors than to those of unrelated inhibitors. The data and code are available at https://github.com/KU-MedAI/DEG2MOL.

## Graphical abstract

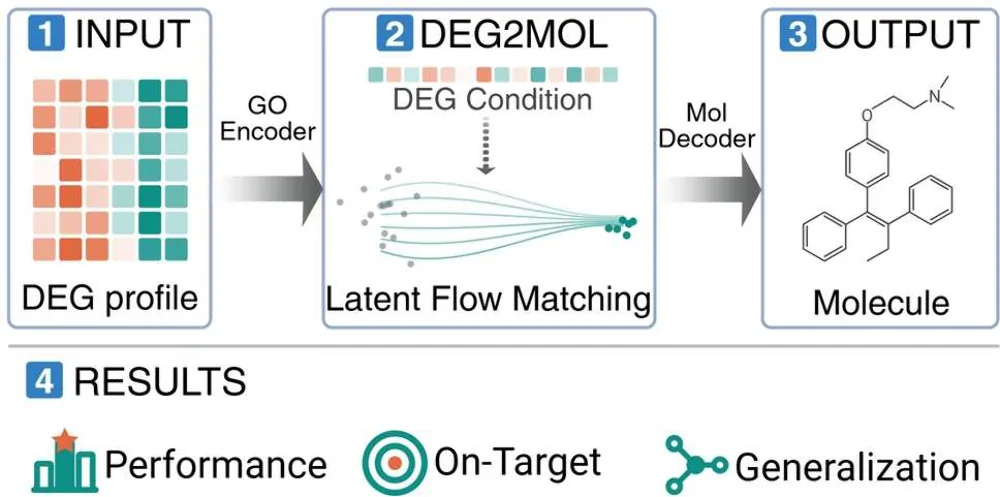

## Introduction

Despite remarkable progress in both experimental and computational methodologies, the development of novel therapeutics remains costly, with high attrition rates.^1,2^ These persistent challenges have motivated a paradigm shift in drug discovery, from traditional experimental approaches such as high-throughput screening and virtual screening of predefined chemical libraries toward deep learning-based *de novo* drug design.^3^ Unlike library-based screening approaches, *de novo* drug design generates molecular structures from scratch, enabling systematic exploration of chemical spaces beyond the reach of existing chemical libraries.^4,5^

In recent years, various generative models have been introduced and applied to *de novo* drug design, including generative adversarial networks (GANs),^6^ variational autoencoders (VAEs),^7^ diffusion models,^8^ and flow matching (FM).^9^ For example, MedGAN^10^ employs the Wasserstein GAN (WGAN) combined with graph convolutional networks to transform random noise into molecular graphs. ScafVAE^11^ leverages a graph-based VAE to generate molecules with optimized properties through scaffold fragment decomposition. Furthermore, the geometry-complete diffusion model (GCDM)^12^ employs geometric diffusion based on graph neural networks (GNNs) to generate structurally stable and large molecules for 3D molecular optimization. In parallel, target-conditioned generative models have been developed in structure-based drug design to generate ligands conditioned on protein targets or binding pockets. For instance, Pocket2Mol^13^ employs an equivariant GNN to sample atoms and bonds conditioned on 3D protein pocket geometry, while TargetDiff^14^ and DiffSBDD^15^ formulate target-aware molecular generation as a protein pocket-conditioned 3D diffusion process. These models improve target-specific molecular generation by incorporating structural information from binding sites but generally require known target structures or predefined binding pockets. Moreover, even when structural information is available, these approaches predominantly optimize physicochemical descriptors such as a quantitative estimate of druglikeness (QED) and logP^16^ and do not explicitly condition generation on disease- or perturbation-relevant biological response profiles. This limits their ability to generate molecules that are guided by functional cellular states.

Transcriptomic profiles offer a compelling alternative, capturing high-dimensional cellular responses to chemical and genetic perturbations as phenotypic readouts for drug design.^17^ This approach aligns with the principles of phenotypic drug discovery (PDD), wherein molecules are designed to elicit desired cellular effects rather than to optimize against predefined target structures or physicochemical objectives.^18^ This direction has been enabled by large-scale, perturbation-induced transcriptomic resources. The Library of Integrated Network-Based Cellular Signatures (LINCS) L1000 project provides over 3 million transcriptional profiles across chemical and genetic perturbations, such as knockdown (KD) and knockout (KO), using the cost-effective L1000 assay, which measures 978 landmark genes to infer the expression of over 10,000 genes.^19^ Additionally, publicly available Perturb-seq^20^ datasets provide scalable, high-resolution transcriptomic readouts of perturbations at single-cell resolution.^21,22^ Together, these resources have motivated deep-learning models that bridge transcriptomic profiles and molecular structures for transcriptome-guided *de novo* drug design.

Several deep learning models have leveraged the LINCS L1000 dataset to bridge transcriptomic profiles and molecular structures; however, existing frameworks face critical limitations from both architectural and data perspectives. GAN-based models, such as the conditional WGAN,^23^ map transcriptional responses to molecules but the reported molecular validity of 8.2% indicates challenges in generating chemically valid outputs. VAE-based frameworks resolve this validity problem by decoding molecules from a learned continuous latent space, and FAME,^24^ BiCEV,^25^ and GxVAEs^26^ have applied this strategy to transcriptome-conditioned generation, reporting validity of 84%, 96%, and above 78%, respectively. The burden of evidence thereby shifts from chemical validity to biological specificity, which all three establish only indirectly: FAME reports a single set-level Fréchet ChemNet Distance to known actives pooled over ten targets under KO conditioning, BiCEV finds mixed correspondence to functional equivalents on held-out knockdown profiles, and GxVAEs relies solely on Tanimoto similarity to known ligands under a random, non-scaffold-aware split. TransGEM,^27^ an autoregressive transformer-based model that generates molecules represented as self-referencing embedded strings (SELFIES)^28^ using cell lines and differential expression values, frequently produces redundant structures due to its deterministic beam-search decoding algorithm. More recently, the graph latent diffusion model (GLDM)^29^ generates molecular graphs through iterative latent denoising, requiring repeated refinement of molecular representations during sampling. GGIFragGPT^30^ employs a Geneformer^31^ conditioned autoregressive decoder that constructs molecules from differentially expressed genes (DEG) profiles by sequentially adding BRICS fragments until an end token is generated, making molecular composition dependent on successive fragment-selection and termination decisions. From a data perspective, most approaches condition generation on post-treatment gene expression profiles, which entangle compound-induced signals with basal cell line identity, and rely on restricted subsets of cell lines, limiting generalizability to unseen biological contexts. Moreover, the reliance on landmark genes rather than the whole transcriptome results in significant information loss, restricting the biological resolution of the learned representations. Furthermore, the absence of scaffold-aware data partitioning permits structural overlap between training and test compounds, obscuring whether models perform genuine *de novo* design.

To address these limitations, we propose DEG2MOL as a biologically grounded conditioning framework that integrates a Gene Ontology (GO)^32^ informed DEG encoder with a frozen, pre-trained scaffold-aware VAE through conditional latent FM^33^ for transcriptome-guided *de novo* drug design. By conditioning molecular generation on DEG profiles rather than post-treatment expression values, DEG2MOL isolates compound-specific transcriptional signals from cell line-specific background expression, providing a cleaner and more transferable biological condition. DEG2MOL utilizes a GO-informed DEG encoder to embed the differential expression levels of 10,280 genes, leveraging the hierarchical structure of GO to incorporate prior biological knowledge into the transcriptomic representation, and generates molecules by learning a velocity field that transforms Gaussian noise into molecular latent vectors within the continuous latent space of a frozen, pre-trained ScafVAE. Classifier-free guidance (CFG)^34^ further strengthens the adherence of generated structures to the given biological condition. In addition, we enforce scaffold-aware data partitioning to enable rigorous evaluation of structural generalization and *de novo* design capability. We evaluate DEG2MOL across compound, KD, and KO-induced, and single-cell Perturb-seq-derived DEG profiles. We further examine representative associations between GO-associated transcriptomic representations and pharmacophore features in generated molecules, conduct illustrative docking analyses of two compound–target pairs, and perform an *in silico* gene expression prediction analysis to assess transcriptomic-response similarity.

## Methods

### Dataset

#### Training Data

We used the LINCS L1000 Level 5 DEG profiles, each representing the differential gene expression values for 12,328 genes quantified as moderated *z*-scores relative to control profiles. We restricted our analysis to profiles measured under a treatment duration of 24 h and a compound concentration of 10 μM. We retained only profiles satisfying both a replicate correlation threshold (*cc*_*q*75 ≥ 0.2) and a self-similarity rank percentile criterion (*pct*_*self*_*rank*_*q*25 ≤ 0.05), referred to as gold signatures. This procedure yielded a dataset of 13,743 samples from 16 distinct cell lines, which served as the foundation for training and evaluating DEG2MOL.

#### Integration of Gene Ontology Hierarchy

To incorporate prior biological knowledge into the DEG autoencoder (AE), we used the hierarchical structure of the GO Biological Process (GO-BP). This hierarchy comprises 16 levels, where lower numerical levels correspond to more general biological processes, and higher numerical levels represent increasingly specific processes. For input features, we selected 10,280 genes common to both the LINCS L1000 and GO-BP data sets.

#### Dataset Partitioning

We evaluated all models under both random- and scaffold-based splits using an 8:1:1 ratio. For the random split, samples were partitioned at random at the level of the individual perturbation profiles. For scaffold-based splitting, Murcko scaffolds were extracted from the SMILES representation of each compound using RDKit,^35^ and compounds sharing identical scaffolds were assigned to the same partition to ensure that no scaffold was shared across training, validation, and test sets, enabling a more stringent evaluation of model generalization to structurally novel compounds.

### Molecular Latent Space Construction

We employed a pre-trained ScafVAE to construct a continuous latent vector of molecular structures (Figure 1A). ScafVAE employs GNN-based encoder *E*_mol_ and decoder *D*_mol_ architectures pre-trained on large-scale molecular datasets, including ChEMBL^36^ and ZINC,^37^ to map molecules into a Gaussian-distributed latent space. By decomposing molecules into core scaffolds and fragments, modeling the scaffold as a structural backbone and the fragments as modular attachments, ScafVAE captures both the global chemical framework and local chemical variability, enabling structured and chemically coherent molecule generation. Our framework integrates the pre-trained ScafVAE parameters without further fine-tuning. For a given molecule *m*, the encoder *E*_mol_ maps it to a latent vector $z_{m}\in R^{64}$ by parameterizing a Gaussian distribution:

1 $$q_{\mathrm{mol}}\left(z_{m}|m\right)=N\left(\mu_{m},\mathrm{diag}\left(\sigma_{m}^{2}\right)\right)$$

**Figure 1:**
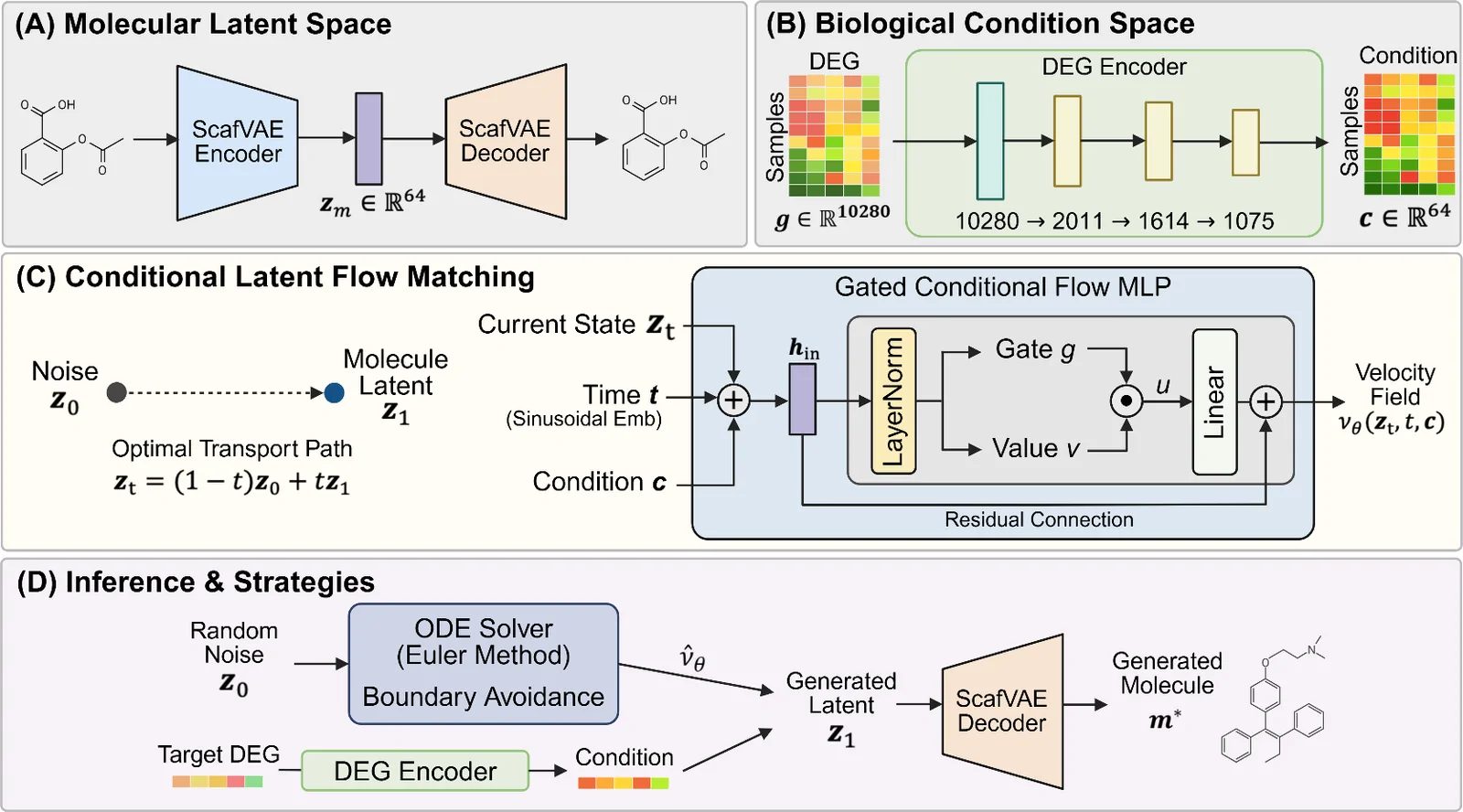
Overall architecture and workflow of DEG2MOL. (A) Molecular latent space: A pre-trained ScafVAE defines a continuous 64-dimensional latent space **z**_*m*_ for molecular structures. (B) Biological condition space: High-dimensional DEG data $g\in R^{10280}$ is embedded into a 64-dimensional condition vector **c** through a Gene Ontology-informed DEG encoder. (C) Conditional latent flow matching: The generative process is modeled as a flow matching problem using an optimal transport path, where a gated conditional flow MLP integrates the current state **z**_*t*_, sinusoidal time embeddings *t*, and the condition **c** via a summation-based fusion to predict the velocity field *v*_*θ*_. (D) Inference and strategies: Starting from random noise **z**_0_, molecules are generated by solving the ordinary differential equation incorporating the target condition c* via classifier-free guidance with boundary avoidance and exponential moving average (EMA). The final molecular structure m* is decoded from the latent vector using the ScafVAE decoder.

The resulting latent space serves as the target distribution for the DEG2MOL generative process, where the FM model learns to transform Gaussian noise into molecular latent vectors that can be decoded by *D*_*mol*_.

### Encoding Biological Condition

To encode transcriptomic perturbation signals as biological conditions for molecular generation, we pre-trained a DEG AE on LINCS L1000 DEG profiles from 16 cell lines. Because the DEG AE learns generalized transcriptomic patterns through unsupervised reconstruction rather than compound-specific associations, it does not require the stringent reliability criteria used to define gold signatures; we therefore pre-trained it on the full available profile set to capture a broader range of DEG patterns. The architecture mirrors the hierarchical GO-BP structure to incorporate prior biological knowledge into the transcriptomic representation.^38^ Following pre-training, we extracted only the encoder (*E*_deg_) to transform the high-dimensional input $g\in R^{10280}$ into a latent vector $c\in R^{64}$ (Figure 1B), which served as the biological condition for the conditional latent FM framework. Further details on the DEG AE architecture are provided in Supporting Information, Figure S1, and the hyperparameter configuration and tuning process for selecting the final architecture are described in Supporting Information, Figure S2.

## Conditional Latent Flow Matching Framework

### Latent Flow Matching and Training Strategies

DEG2MOL employed FM to learn the conditional mapping from the DEG condition **c** to the molecular latent vector **z**_*m*_ (Figure 1C). In our framework, **z**_*m*_ obtained from the pre-trained ScafVAE encoder is defined as the target state at *t* = 1 (i.e., **z**_1_ = **z**_*m*_), and **z**_0_ represents a sample from the Gaussian prior $p_{0}=N\left(0,I\right)$ . We defined a time-dependent probability path *p*_*t*_ that connects a Gaussian prior $p_{0}=N\left(0,I\right)$ to the target molecular distribution *p*_1_. For each mini-batch, we solved a linear assignment problem using the optimal transport (OT) path to pair noise samples **z**_0_ with target samples **z**_1_, minimizing the total movement cost. The intermediate state **z**_*t*_ at time *t* ∈ [0, 1] is defined by linear interpolation:

2 $$z_{t}=\left(1-t\right)z_{0}+tz_{1}$$

The target velocity field *u*_*t*_ is constant:

3 $$u_{t}\left(z_{t}|z_{0},z_{1}\right)=z_{1}-z_{0}$$

The network *v*_*θ*_(**z**_*t*_, *t*, **c**) is trained to approximate this velocity by minimizing the FM loss:

4 $$L_{FM}=E_{t,z_{0},z_{1},c}\left[{∥v_{\theta }\left(z_{t},t,c\right)-\left(z_{1}-z_{0}\right)∥}^{2}\right]$$

We applied CFG by randomly replacing the condition **c** with a null token ⌀ (zero vector) with probability *P*_drop_ = 0.3. This training scheme enables the model to learn conditional and unconditional distributions simultaneously:

5 $$c_{train}=\{\begin{array}{ll} c & \mathrm{with probability}1-P_{\mathrm{drop}}\\ ⌀ & \mathrm{with probability}P_{\mathrm{drop}} \end{array}$$

We also employed a boundary avoidance strategy^39^ to prioritize more informative intermediate trajectories and enhance stability. Specifically, the time step *t* is sampled via a sigmoid transformation of a standard normal variable z∼N(0,1) such that *t* = σ­(*z*) · (1 – 2ϵ) + ϵ, where σ(·) is the sigmoid function and ϵ = 10^–5^ keeps *t* within the open interval (0, 1).

To enhance the robustness and consistency of molecular generation, we applied an exponential moving average (EMA)^40^ to the model weights θ with a decay rate β = 0.999. At each training step *k*, the EMA weights are updated as

6 $$\theta_{EMA}^{\left(k\right)}=\beta \theta_{EMA}^{\left(k-1\right)}+\left(1-\beta \right)\theta_{online}^{\left(k\right)}$$

where θ_online_ represents the online parameters. These accumulated EMA weights are subsequently utilized for all inference tasks.

### Network Architecture

The velocity field *v*_θ_ integrates the current state **z**_*t*_, time *t*, and condition **c** through a summation-based fusion approach.

#### Input Projection and Conditioning

Inputs are first embedded into 512 dimensions. Specifically, **x**_proj_ is derived via a linear transformation of $z_{t}\in R^{64}$ . The time *t* is transformed using sinusoidal embeddings with SiLU activation to obtain **t**_emb_, and the biological condition **c** is processed through layer normalization and a linear projection to obtain **c**_emb_. These features are integrated via element-wise summation:

7 $$h_{\mathrm{in}}=x_{\mathrm{proj}}+t_{\mathrm{emb}}+c_{\mathrm{emb}}$$

#### Gated MLP Blocks

The integrated feature **h**_*in*_ is processed using six-layer gated multi-layer perceptron (MLP) blocks, with **h** ^(0)^ = **h**_in_. For the input **h** ^(*l*)^ to the *l*-th block:

8 $${ĥ}^{\left(l\right)}=\mathrm{Layer Norm}\left(h^{\left(l\right)}\right)$$

9 $$g^{\left(l\right)}=W_{\mathrm{gate}}{ĥ}^{\left(l\right)}+b_{\mathrm{gate}},,v^{\left(l\right)}=W_{\mathrm{in}}{ĥ}^{\left(l\right)}+b_{in}$$

10 $$u^{\left(l\right)}=SiLU\left(g^{\left(l\right)}\right)⊙v^{\left(l\right)}$$

11 $$h^{\left(l+1\right)}=h^{\left(l\right)}+\left(W_{\mathrm{out}}u^{\left(l\right)}+b_{\mathrm{out}}\right)$$

where ⊙ denotes element-wise multiplication. The output projection **W**_out_ is initialized to zero to stabilize the initial training phase, ensuring the block initially acts as an identity function via the residual connection.

### Inference

During the inference phase, a target DEG profile g* is first converted into a condition vector c* = *E*_deg_(g*) (Figure 1D). Following CFG, we use a guided velocity field with guidance scale *s* = 3.0:

12 $${\mathrm{v̂}}_{\theta }\left(z_{t},t,c^{*}\right)=v_{\theta }(z_{t},t,⌀+s\cdot \left(v_{\theta }\left(z_{t},t,c^{*}\right)-v_{\theta }(z_{t},t,⌀\right)$$

Starting from random noise $z_{0}∼N\left(0,I\right)$ , we initialize the inference trajectory as ${ẑ}_{t_{0}}=z_{0}$ and solve the ordinary differential equation (ODE) through *N* = 100 discrete time steps using the Euler method:

13 $${ẑ}_{t_{i+1}}={ẑ}_{t_{i}}+\Delta t\cdot {\mathrm{v̂}}_{\theta }\left({ẑ}_{t_{i}},t_{i},c^{*}\right)$$

where ${ẑ}_{t_{i}}$ denotes the inference-time latent state after *i* Euler updates, with step size Δ*t* = 1/*N* and *t*_*i*_ = *i*/*N* for *i* = 0, ..., 99. The final state ${ẑ}_{1}$ is then decoded by the pre-trained ScafVAE decoder *D*_mol_:

14 $$m^{*}=D_{\mathrm{mol}}\left({ẑ}_{1}\right)$$

### Implementation Details

#### Baseline Model Evaluation Protocol

We trained and evaluated all baseline models using LINCS L1000 data prepared with the same filtering criteria and split assignments as DEG2MOL, ensuring that performance differences reflect model design rather than data partitioning. The only exception was in the L1000 data level and gene coverage, which varied across models in accordance with their original formulations. Specifically, FAME, GxVAEs, and TransGEM, which operate on gene expression profiles, used Level 3 data, where duplicate signatures from the same experimental condition were averaged, whereas the remaining models used Level 5 differential expression profiles consistent with DEG2MOL. Regarding gene coverage, FAME, GxVAEs, TransGEM, and GGIFragGPT were applied to 978 landmark genes following their original designs, while DEG2MOL utilized 10,280 genes. These differences reflect each model’s architectural design choices rather than experimental confounds introduced by our evaluation protocol. Each model was trained with five random seeds, and evaluation metrics were calculated separately for each seed and reported as the mean ± standard deviation across the five seed-level values.

#### DEG2MOL

We optimized the model using the AdamW optimizer with an initial learning rate of 0.0002, weight decay of 10^–5^, and batch size of 128. The learning rate was scheduled using a Cosine Annealing Warm Restarts strategy (*T*_0_ = 10, *T*_mult_ = 2). All models were trained on a computing machine equipped with an Intel Xeon Gold 6148 CPU, 256 GB memory, and an NVIDIA A100 PCIe 80 GB GPU.

### Evaluation Metrics and Statistical Analysis

#### Molecular Generation Metrics

For each input DEG profile, each model independently generated 100 molecules, and all metrics described below were computed over these generated sets. Validity, novelty, and uniqueness were defined as the proportions of RDKit-valid molecules, molecules absent from the training set, and nonredundant valid molecules, respectively. To provide a more rigorous evaluation of molecular quality, we adopted cascaded metrics,^41^ Val + Uniq = Validity × Uniqueness and Val + Uniq + Novel = Validity × Uniqueness × Novelty, that sequentially apply validity, uniqueness, and novelty. Unlike conventional frameworks that compute uniqueness and novelty only on valid molecules, cascaded metrics penalize invalid structures across subsequent metrics. Internal diversity (IntDiv) was calculated as one minus the mean pairwise Tanimoto similarity, and scaffold diversity (ScafDiv) was calculated analogously using Murcko scaffolds. For ECFP6,^42^ MACCS Keys,^43^ and Fraggle similarity, the maximum similarity of each generated molecule to the condition-relevant reference compounds was calculated and averaged across generated molecules. ECFP6 and MACCS similarity used 1024-bit radius-3 fingerprints and 166-bit keys, respectively. QED and synthetic accessibility (SA) scores were averaged across evaluated molecules, whereas AutoDock Vina docking scores^44^ were summarized by the median for each target protein.

#### Overall Model Ranking

Within each data split, models were ranked using the five-seed means of validity, uniqueness, novelty, ECFP6, MACCS, and Fraggle similarity rounded to four decimal places. Rank 1 indicated the best value, ties received their midrank, and the six ranks were summed; the random and scaffold split sums were then combined with equal weight to obtain the overall rank. IntDiv and ScafDiv were excluded as ranking criteria because low diversity may indicate mode collapse while high diversity may reflect condition-irrelevant chemical space exploration, whereas the cascaded metrics were excluded to avoid duplicating validity, uniqueness, and novelty.

#### GO-Aligned Node Activation and Pharmacophore Feature Analysis

First-layer GO-aligned node activations were extracted from *E*_deg_ for each test set sample and standardized per node against the mean and standard deviation of all training set samples. Each compound condition was then compared against all remaining compound conditions within the test set. GO terms were ranked by the mean activation difference, and between-condition differences were assessed using Cohen’s *d*,^45^ Welch’s *t*-test,^46^ and permutation test,^47^ followed by Benjamini–Hochberg (BH) correction.^48^ Representative GO terms were selected based on concordant ranks and effect directions across both sets and BH-adjusted significance.

Pharmacophore features were defined using a curated set of 53 patterns derived from the RDKit functional-group hierarchy and quantified for each DEG profile as the proportion of valid generated molecules containing each feature. Pharmacophore features showing compound-specific enrichment with concordant effect directions across the test set were retained for representative analysis. Associations between GO-aligned node activation and pharmacophore prevalence were quantified using Spearman’s ρ with BH correction. LOWESS curves^49^ with 95% confidence intervals were used for visualization.

#### shRNA KD and CRISPR KO Evaluations

For each target, all valid molecules generated from the associated KD or KO DEG profiles were pooled, and their maximum ECFP6 Tanimoto similarity to the reference inhibitors of that target was computed. Reference inhibitors were collected from the ExCAPE database.^50,51^ Top-*k* (*k* = [1, 10, 100]) score was calculated as the mean of the highest *k* similarity scores within a target’s pool. For model comparisons, per-target Top-*k* scores were averaged across the five random seeds and compared across shared targets using one-sided paired Wilcoxon signed-rank tests.^52^ The resulting *p*-values across the KD and KO Top-*k* comparisons were adjusted using the BH correction and reported as *q*-values.

#### In Silico Analysis of Predicted Compound-Induced Expression Profiles

Compound-induced expression profiles were predicted using the cell-type-specific drug perturbation responses predictor (CRISP)^53^ and converted into cell-line-specific, control-standardized *z*-scores across 5000 genes. For each cell line–target combination, the CRISP-predicted profiles of all DEG2MOL-generated molecules were aggregated into a single consensus profile by taking the gene-wise median across molecules. For each cell line, ten target-matched reference molecules were selected for each of AKT1, MTOR, and PIK3CA, while a comparator group of ten molecules was selected for each of AURKB, HDAC1, and CTSK.

Profile similarity was quantified using Spearman’s ρ across all 5000 genes and the signed Jaccard index. For the signed Jaccard analysis, the 50 genes with the largest absolute *z*-scores were selected independently from each profile, with the sign retained to represent the predicted direction of regulation. For each target-matched reference molecule, its similarity to the corresponding DEG2MOL consensus profile was paired with its mean similarity to the 30 non-target-matched comparator molecules. Paired differences were evaluated using one-sided exact Wilcoxon signed-rank tests, and the resulting *p*-values were adjusted using the BH procedure to obtain *q*-values.

## Results

DEG2MOL was evaluated across four experimental settings spanning compound-induced, shRNA-KD-induced, CRISPR-KO-induced, and Perturb-seq DEG profiles. For compound-induced profiles, we assessed molecular generation performance under random and scaffold splits, associations between GO-informed encoder activations and pharmacophore features, and molecular docking analyses. We then evaluated transfer to genetic perturbations and Perturb-seq data using structural similarity to known target inhibitors and CRISP-predicted transcriptional profile similarity.

### Molecular Generation from Compound-Induced DEG Profiles

First, we assessed the performance of DEG2MOL in generating chemically valid and condition-relevant molecules from the compound-induced DEG profiles. As shown in Table 1, while most models achieved comparable performance on individual metrics, DEG2MOL achieved the top rank within each split when assessed by rank-sum aggregation of six metrics (validity, uniqueness, novelty, ECFP6, MACCS, and Fraggle). Under the random split, DEG2MOL achieved the highest uniqueness (0.7743 ± 0.0225), the highest cascaded scores (%V+ U, 0.7718 ± 0.0224; %V + U + N, 0.7699 ± 0.0224), and the highest scores for all three structural-similarity metrics: ECFP6 (0.4277 ± 0.0075), MACCS (0.7959 ± 0.0015), and Fraggle (0.6942 ± 0.0040). The mean ECFP6 similarity of DEG2MOL was approximately 1.7 times that of the strongest baseline, GGIFragGPT (0.4277 vs 0.2456). In contrast, TransGEM produced almost entirely redundant outputs under both splits (uniqueness 0.0006 ± 0.0000 and 0.0004 ± 0.0000), as expected for deterministic beam-search decoding used by that model. Under the scaffold split, where test compounds contained Murcko scaffolds not observed during training, DEG2MOL retained the highest cascaded scores (%V + U, 0.8047 ± 0.0194; %V + U + N, 0.8040 ± 0.0194) and the highest ECFP6, MACCS, and Fraggle scores of 0.2214 ± 0.0039, 0.6806 ± 0.0030, and 0.6291 ± 0.0017, respectively. These results suggest that DEG2MOL continues to generate molecules structurally similar to condition-relevant reference compounds even when the test scaffolds were unseen during training. Complete per-metric ranks are provided in Supporting Information, Figure S3 .

**Table 1:**
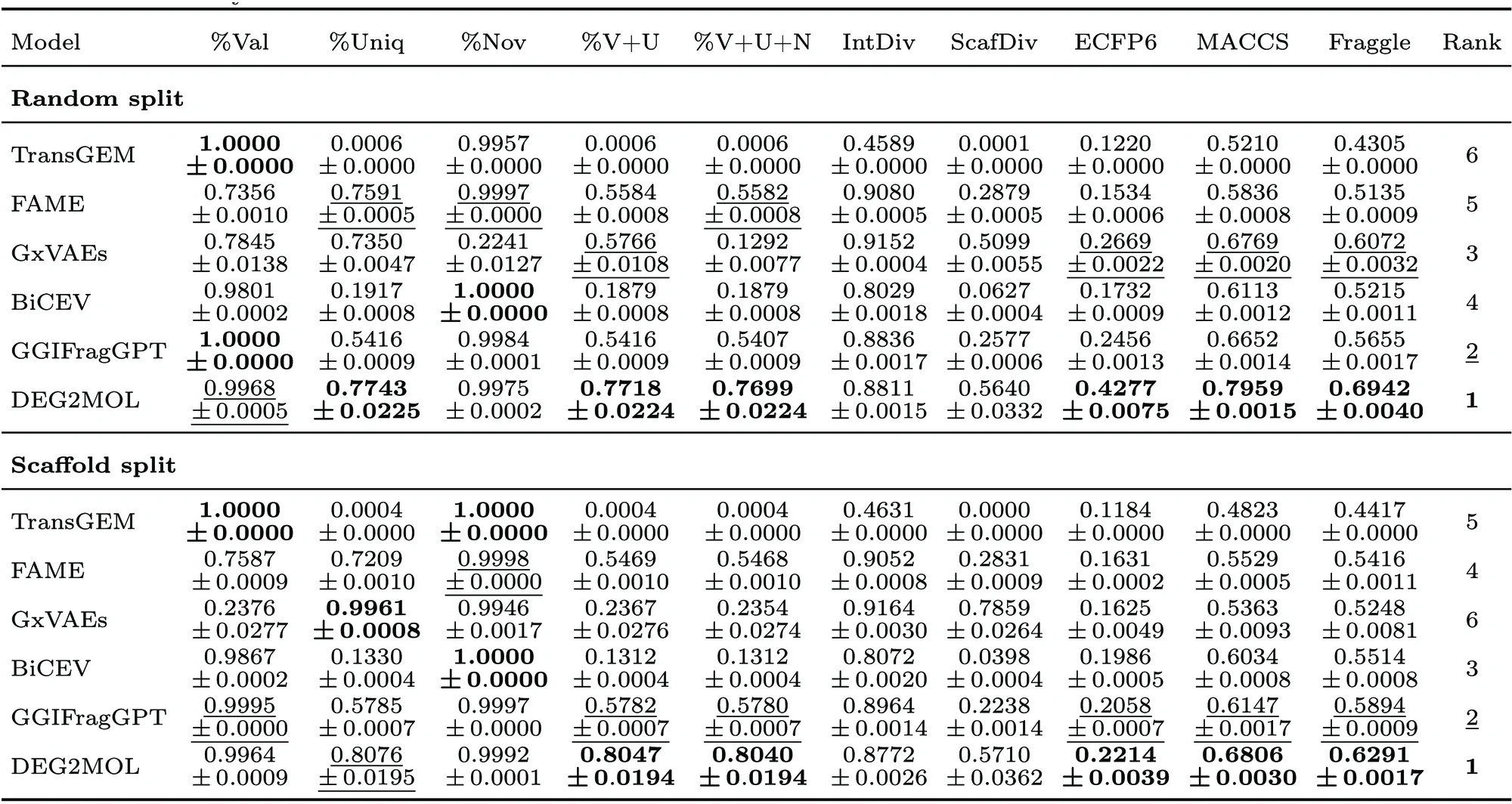
Comparison of Molecular Generation Performance between DEG2MOL and Baseline Models under Random and Scaffold Split Settings^a^

a For each DEG profile, 100 molecules were generated per seed, and values are reported as mean ± standard deviation across five generation seeds. Rank was determined by rank-sum aggregation of %Val, %Uniq, %Nov, ECFP6, MACCS, and Fraggle. Bold and underline denote the best and second-best values within each split, respectively. Abbreviations: IntDiv, Internal Diversity; ScafDiv, Scaffold Diversity.

To verify that the generative model produces condition-specific molecular latent vectors, we computed pairwise cosine similarities among molecular latent vectors generated from compound-induced DEG profiles in the test set, comparing pairs from the same compound across different cell lines against pairs from distinct compounds (Figure 2). Latent vectors from the same compound showed significantly higher cosine similarity than those from different compounds, indicating that DEG2MOL produces distinct molecular latent vectors in response to different DEG conditions. Furthermore, to examine whether this compound specificity extends to the global latent space, we visualized the UMAP projection of both train- and test-set latent vectors and found that the same compound consistently occupied the same region across splits (Supporting Information S4).

**Figure 2:**
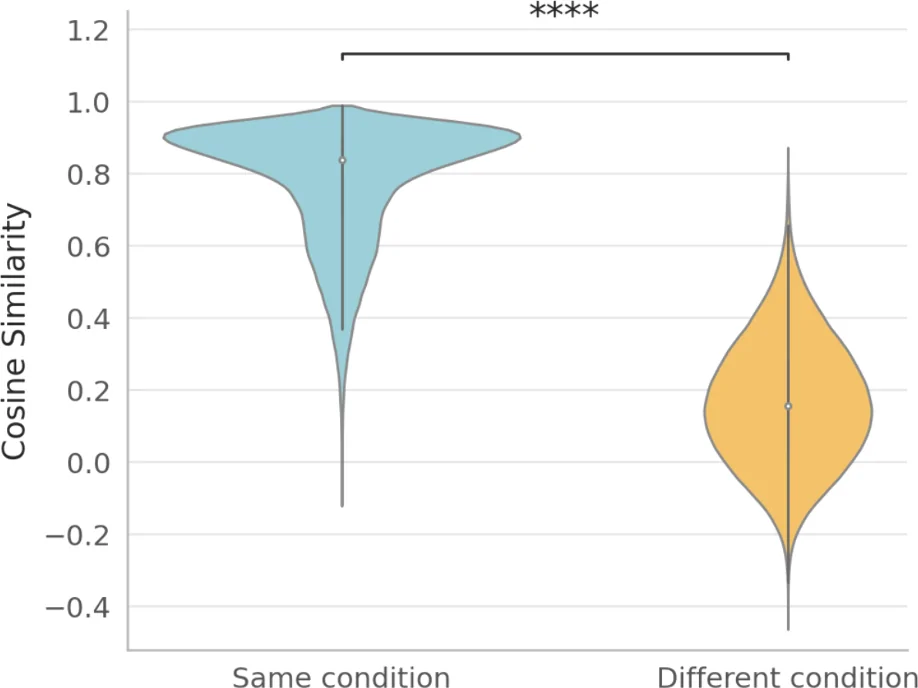
Pairwise cosine similarity distributions of molecular latent vectors generated from compound-induced DEG profiles in the test set. The ”Same condition” distribution represents similarities among latent vectors corresponding to the same compound across different cell lines, while the ”Different condition” distribution represents similarities between latent vectors from distinct compounds. ****p < 0.0001, Mann-Whitney U test.

### Associations between GO-Aligned Node Activations and Pharmacophore Features of Molecules Generated from Compound-Induced DEG Profiles

To examine whether biological signals represented by the GO-informed DEG encoder were associated with chemical features of the generated molecules, we performed a GO–pharmacophore analysis under the random split. Vorinostat and bortezomib were selected as representative cases. (Figure 3). For each compound, we selected the highest-ranked compound-associated GO term and the compound-relevant pharmacophore with the highest prevalence among molecules generated from the corresponding DEG profiles. We then evaluated the association between GO-aligned node activation and pharmacophore prevalence across all compound conditions.

**Figure 3:**
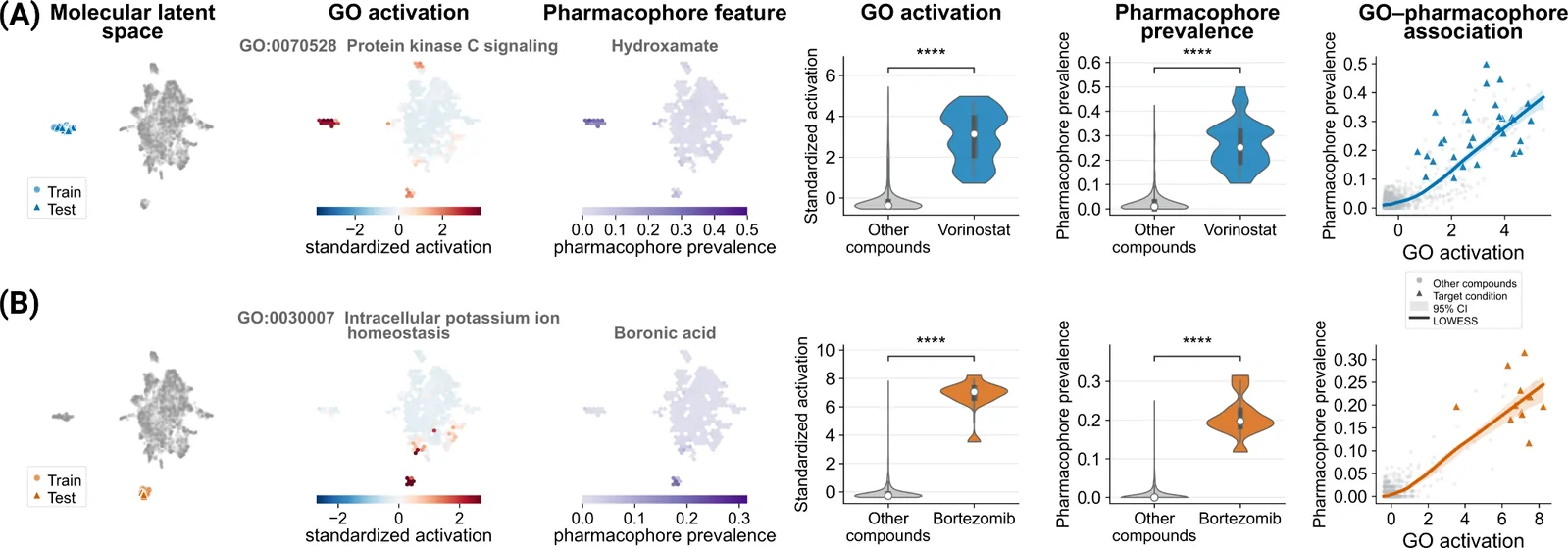
Representative associations between GO-informed DEG encoder activations and pharmacophore features in generated molecules for vorinostat and bortezomib DEG profiles. Panels from left to right show the two-dimensional molecular latent vector, standardized GO-aligned node activation, pharmacophore prevalence, group-wise comparisons with the other compound conditions, and the association between node activation and pharmacophore prevalence. (A) Vorinostat analysis based on protein kinase C signaling (GO:0070528) and the hydroxamate pharmacophore. (B) Bortezomib analysis based on intracellular potassium ion homeostasis (GO:0030007) and the boronic-acid pharmacophore. Colored circles and triangles denote training- and test-set molecular latent vectors for the representative compound, respectively, whereas gray points represent latent vectors from the other compound conditions. Violin plots show the distributions for the target and other compound conditions (****q < 0.001). Association plots show LOWESS fits with 95% confidence intervals.

For vorinostat (Figure 3A), the selected pair comprised protein kinase C signaling (GO:0070528) and the hydroxamate pharmacophore. Hydroxamate was present in 26.6% of molecules generated from vorinostat-induced DEG profiles, compared with 2.8% under the other compound conditions (*d* = 3.45, *q* = 1.0 × 10^–13^). Both features have established relevance to vorinostat: PKC signaling has been reported as a downstream adaptive response to vorinostat exposure,^54^ whereas the hydroxamate group mediates zinc binding within HDAC active sites.^55^ Linking the two observations, GO:0070528 activation was positively associated with hydroxamate prevalence across compound conditions (*ρ* = 0.425, ; *q* = 9.6 × 10^–58^). This association is also biologically plausible in a broader chemical context, as PKC-dependent responses have been reported for trichostatin A, another hydroxamate HDAC inhibitor, and deferoxamine, a hydroxamate-containing iron chelator.^56,57^

For bortezomib (Figure 3B), the selected pair comprised intracellular potassium ion homeostasis (GO:0030007) and the boronic-acid pharmacophore.GO:0030007 showed markedly higher activation under bortezomib than under the other compound conditions (*d* = 9.479, ; *q* = 2.45 × 10^–8^), while boronic acid was present in 21.3% of molecules generated from bortezomib-induced DEG profiles, compared with 0.65% under the other compound conditions. The biological relevance of the GO term is supported indirectly by clinical evidence of bortezomib-associated sensory-axon membrane depolarization, for which altered potassium handling has been proposed as a possible contributor.^58^ Separately, the boronic-acid group is directly involved in bortezomib binding to the proteasome.^59^ GO:0030007 activation was positively associated with boronic-acid prevalence across compound conditions (*ρ* = 0.349, ; *q* = 4.38 × 10^–38^). This association can also be placed in a broader chemical context, as phenylboronic acid has been shown to modulate several Kv7 potassium-channel complexes.^60^

Together, these representative cases show that compound-associated GO-aligned node activations covaried with the prevalence of pharmacophore features relevant to the corresponding compounds. These findings support a condition-level correspondence between the biological representations captured by the GO-informed DEG encoder and the chemical features of the generated molecules without implying a direct or causal relationship between individual GO terms and pharmacophore generation.

### Illustrative Molecular Docking Simulation from Compound-Induced DEG Profiles

Since compound-induced DEG profiles reflect transcriptomic responses to a known drug, molecules generated from such profiles are expected to share similar biological activity with the original compound, including binding to the same target protein. We therefore employed molecular docking as an indirect yet principled means of evaluating whether the generated molecules recovered docking poses and interaction patterns consistent with the reference ligand. As representative examples, molecules generated from the compound-induced DEG profiles of vorinostat and gefitinib were docked to their original target proteins, HDAC2 (PDB 4LXZ) and EGFR (PDB 2ITY), respectively.

To compare docking scores across targets, we conducted cross-docking experiments in which both reference compounds and generated molecules were docked against both HDAC2 and EGFR crystal structures regardless of their intended target. Docking was repeated across the five generation seeds, and Figure 4 A reports the mean and standard deviation of the seed-level median docking scores. The generated molecules obtained more favorable docking scores at their intended targets than at the other target (*p* < 0.001, Mann–Whitney U test). Vorinostat-derived molecules showed a median docking score of −7.577 ± 0.068 kcal/mol against HDAC2 compared to −7.178 ± 0.049 kcal/mol against EGFR, while gefitinib-derived molecules achieved −8.108 ± 0.234 kcal/mol against EGFR compared to −7.584 ± 0.199 kcal/mol against HDAC2. These scores were within a comparable range to those of the original reference compounds (vorinostat: −7.49 kcal/mol at HDAC2; gefitinib: −7.69 kcal/mol at EGFR).

**Figure 4:**
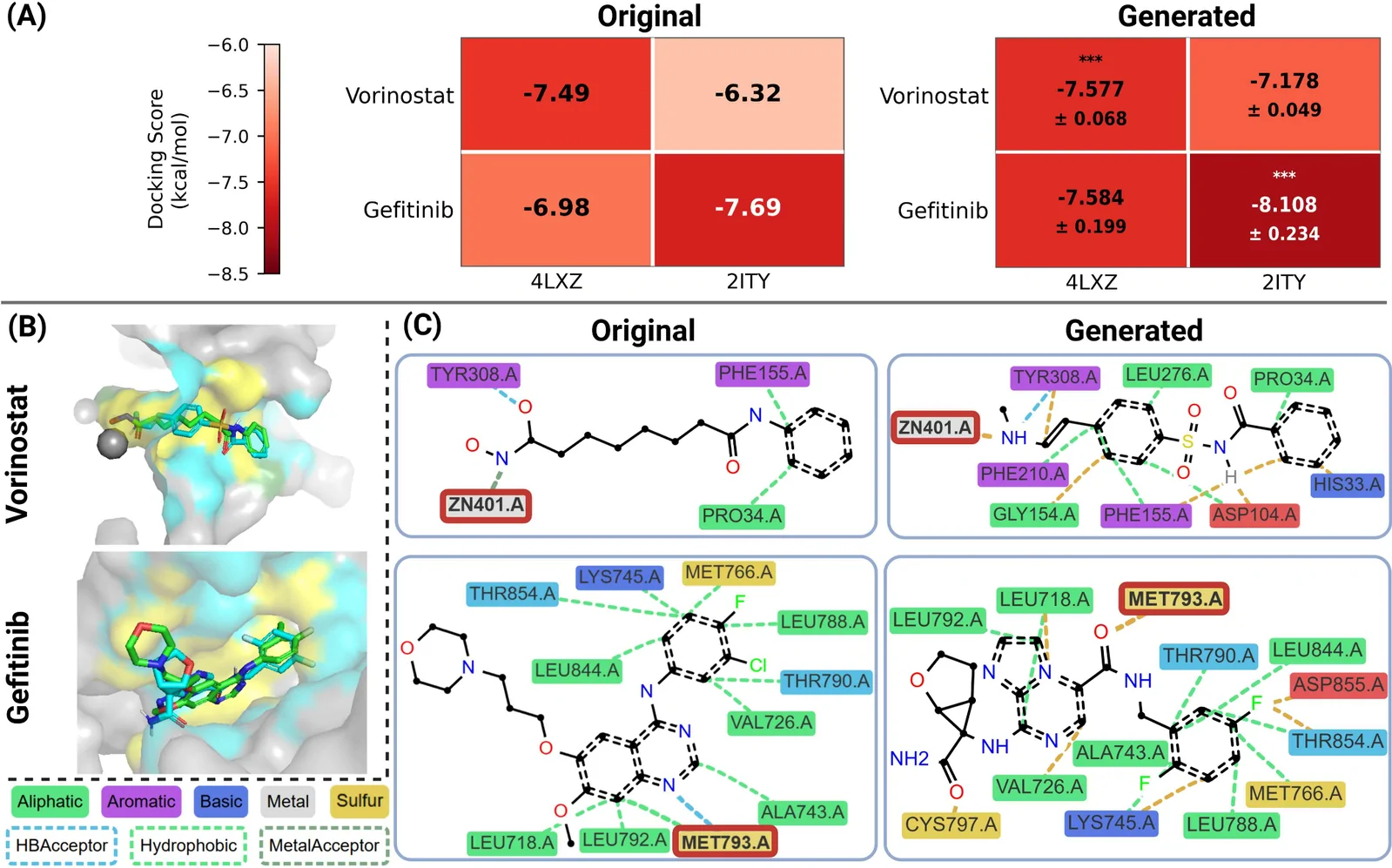
Molecular docking analysis of molecules generated from compound-induced DEG profiles. (A) Cross-docking heatmaps showing median docking scores (kcal/mol) of the original reference compounds (left) and the generated molecules (right) against HDAC2 (PDB 4LXZ) and EGFR (PDB 2ITY) crystal structures. For generated molecules, values are reported as the mean ± standard deviation of the seed-level median docking scores across five generation seeds. Asterisks denote statistically significant differences in docking score distributions for generated molecules between the intended target and the cross target as determined by the Mann–Whitney U test (***p < 0.001). (B) Superimposed binding poses of the reference compound from the co-crystal structure (green) and a generated molecule (cyan) within the binding pocket rendered as a molecular surface. Contacts of the generated molecule are highlighted in cyan, with overlapping contacts shared with the reference compound further highlighted in yellow. (C) Two-dimensional interaction diagrams depicting protein–ligand contacts for the original reference compound (left) and the generated molecule (right). Key catalytic or binding-critical residues reported in the literature are highlighted with red boxes.

To further assess whether the generated molecules reproduced the same binding interactions as the reference drugs, we performed interaction fingerprint analysis of the docking poses using the ProLIF library.^61^ Structural studies have identified critical binding determinants for each target: vorinostat coordinates with the catalytic zinc ion (Zn^2+^),^62^ and gefitinib is anchored by a hydrogen bond to Met793.^63^ Key interactions were preserved in 45.6% and 68.0% of the vorinostat- and gefitinib-derived molecules, respectively. Among these, the top-ranked candidates selected by the Chamfer distance exhibited highly similar binding poses with planar scaffolds closely aligned to those of the reference ligands (Figure 4B) and showed interaction profiles nearly identical to those of the reference compounds (Figure 4C). For these two representative pairs, the generated molecules recovered the reference-associated contacts and docking poses.

### Molecular Generation from LINCS L1000 shRNA Knockdown-Induced DEG Profiles

Although DEG2MOL was trained exclusively on compound-induced DEG profiles, we evaluated its performance on genetic perturbation profiles to assess the cross-domain generalizability. We generated molecules based on LINCS L1000 shRNA KD-induced DEG profiles across a selected set of eight target genes: AKT1, AKT2, AURKB, CTSK, EGFR, HDAC1, MTOR, and PIK3CA. Since KD-induced DEG profiles reflect the transcriptional consequences of suppressing a specific gene, we therefore used structural similarity to known inhibitors as a proxy for target relevance.

As shown in Table 2, DEG2MOL achieved higher target-averaged Top-1 and Top-10 ECFP6 similarities than GGIFragGPT across the eight target genes (Top-1:0.916 vs. 0.742; Top-10:0.810 vs. 0.662), with higher values for seven of the eight targets at both levels (Top-1: seven higher, one tie at EGFR; Top-10: seven higher, one lower at EGFR). Both comparisons were supported by one-sided paired Wilcoxon signed-rank tests across the eight shared targets (Δ = +0.174 and +0.148; *q* = 0.031 for both). DEG2MOL reached a Top-1 similarity of 1.000 ± 0.000 for AKT1, AKT2, AURKB, and MTOR, tied with GGIFragGPT at 1.000 ± 0.000 for EGFR, and was higher for CTSK, HDAC1, and PIK3CA. The target-averaged Top-100 similarity was only slightly higher for DEG2MOL (0.536 vs. 0.515) and did not reach significance after correction (Δ = +0.021; *q* = 0.230), with GGIFragGPT higher for EGFR, HDAC1, and PIK3CA.

**Table 2:**
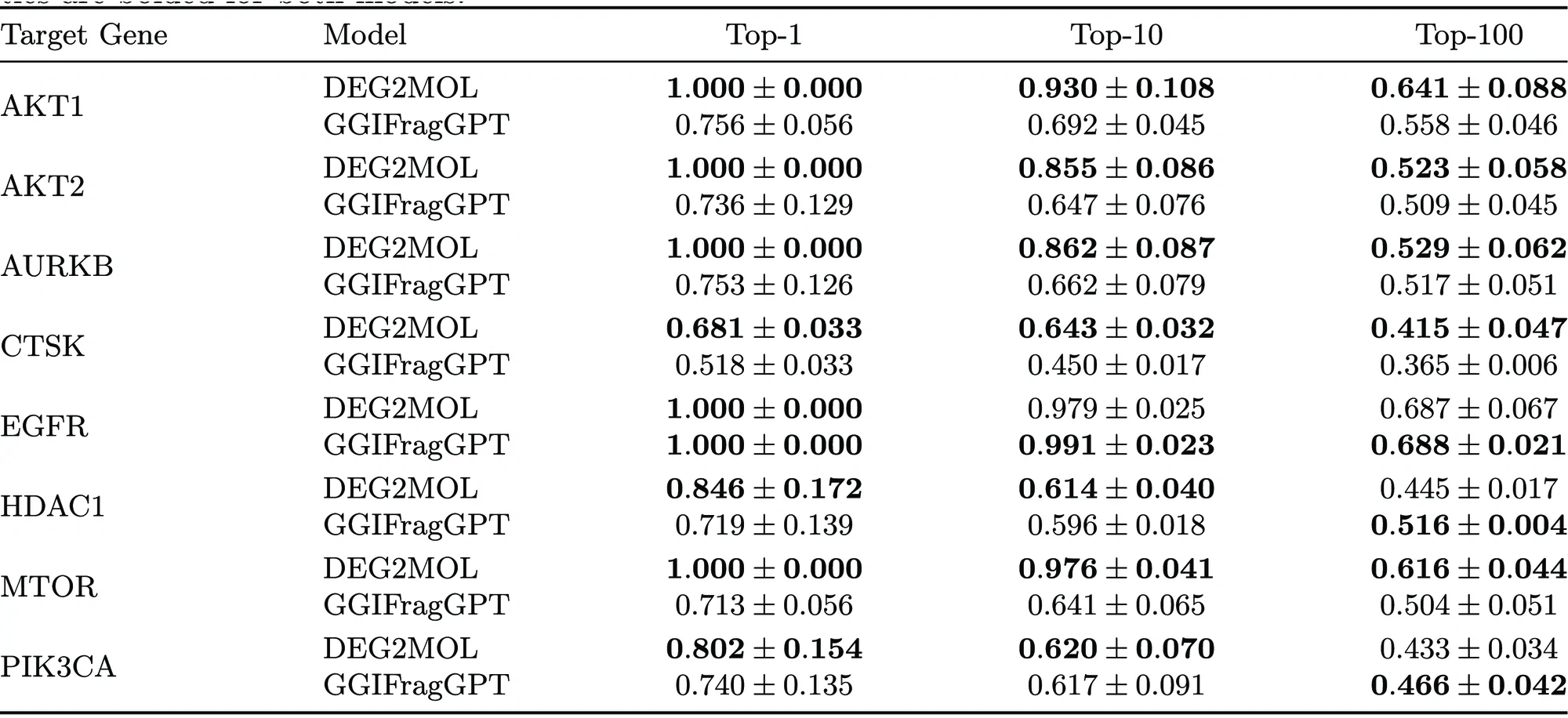
Molecular Generation Performance Based on LINCS L1000 shRNA Knockdown-Induced DEG Profiles^a^

a For each DEG profile, 100 molecules were generated, and molecules associated with the same target gene were pooled for evaluation. Top-*k* represents the mean of the *k* highest ECFP6 Tanimoto similarities between generated molecules and known inhibitors of the corresponding target. Values are reported as mean ± standard deviation across five generation seeds. Bold indicates the higher mean for each target and metric; ties are bolded for both models.

To examine the targets at which GGIFragGPT exceeded DEG2MOL, we compared the physicochemical properties of the generated molecules with those of the corresponding reference inhibitors. Molecules generated by DEG2MOL overlapped more closely with the reference distributions of molecular weight, logP, QED, TPSA, and heavy-atom count than those generated by GGIFragGPT (Supporting Information, Figure S5). Restricting each target-level candidate pool to molecules within the reference molecular-weight window (mean ± 1 standard deviation) retained 3.5–4.5 times more candidates for DEG2MOL and yielded higher target-averaged Top-1, Top-10, and Top-100 similarities for DEG2MOL at all eight targets (*q* = 0.008). Visualization of representative molecules for AKT1, CTSK, HDAC1, and MTOR confirmed that the generated structures retained key structural features of the reference inhibitors and showed Tanimoto similarities comparable to or higher than those of GGIFragGPT (Figure 5). These results indicate that DEG2MOL transferred to the KD-induced profiles evaluated here despite being trained exclusively on compound-induced data.

**Figure 5:**
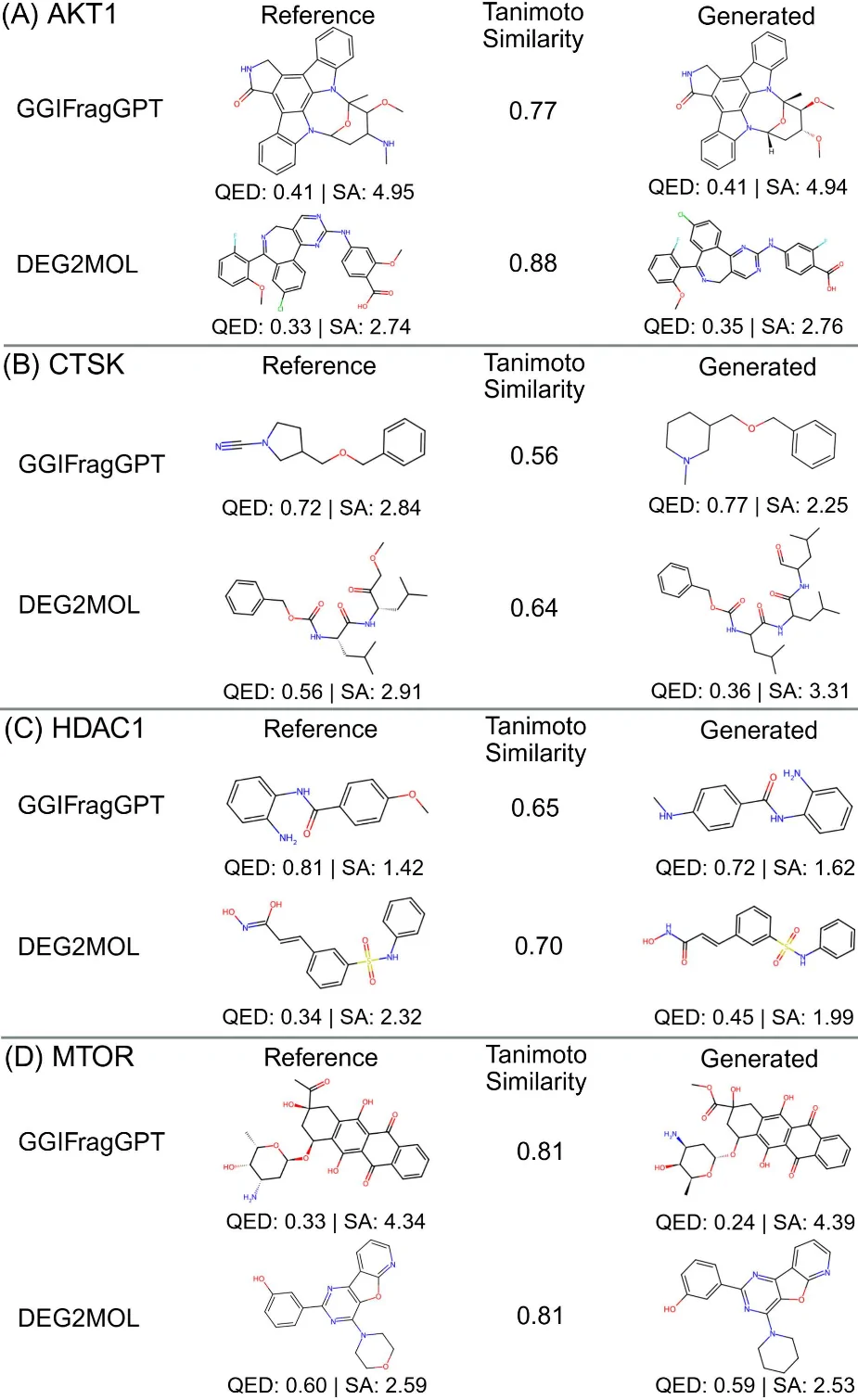
Visualization of molecules generated from LINCS L1000 knockdown-induced DEG profiles. For each therapeutic target, (A) AKT1, (B) CTSK, (C) HDAC1, (D) MTOR, both GGIFragGPT (top) and DEG2MOL (bottom) generated 100 molecules. For each panel, the molecule that achieved the maximum ECFP6 Tanimoto similarity to the known reference inhibitor curated from ExCAPE is shown. QED and SA scores are labeled for both reference and generated molecules.

### Molecular Generation from LINCS L1000 CRISPR Knockout-Induced DEG Profiles

We further evaluated DEG2MOL on LINCS L1000 CRISPR KO-induced DEG profiles across seven target genes: AKT1, AURKB, CTSK, EGFR, HDAC1, MTOR, and PIK3CA. Generation and evaluation followed the same protocol as the KD analysis, and AKT2 was excluded because no AKT2-targeting KO profiles were available in the LINCS L1000 data set.

As shown in Table 3, DEG2MOL achieved a higher target-averaged Top-1 ECFP6 similarity than GGIFragGPT across the seven target genes (0.800 vs. 0.655), with higher values for six targets and a tie at EGFR; the comparison was supported by a one-sided paired Wilcoxon signed-rank test across the shared targets (Δ = +0.145; *q* = 0.031). The Top-10 comparison also favored DEG2MOL (0.635 vs. 0.542; six targets higher, HDAC1 lower) but did not reach significance after correction (Δ = +0.093; *q* = 0.082). At Top-100, GGIFragGPT obtained a slightly higher target average (0.418 vs. 0.410; higher for AURKB, EGFR, HDAC1, and PIK3CA), and the difference was not significant (Δ = −0.008; *q* = 0.656).

**Table 3:**
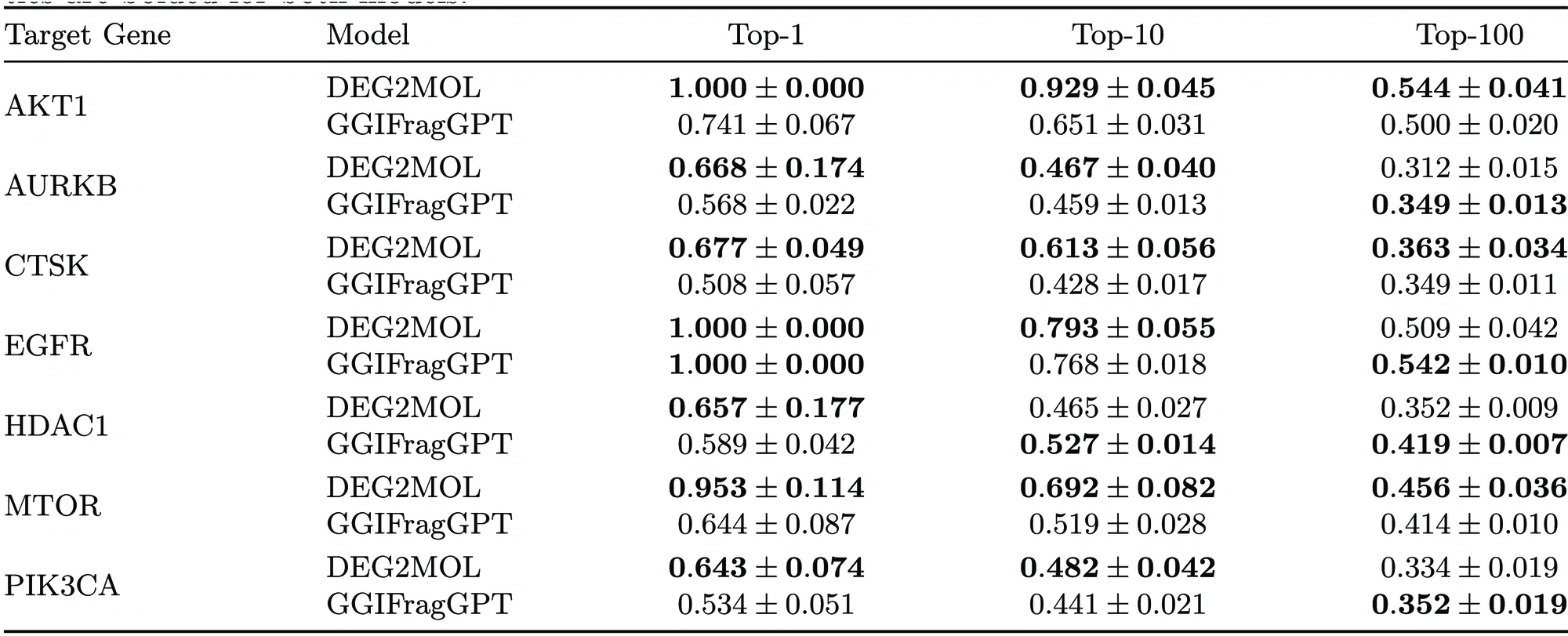
Molecular Generation Performance Based on LINCS L1000 CRISPR Knockout-Induced DEG Profiles^a^

a For each DEG profile, 100 molecules were generated, and molecules associated with the same target gene were pooled for evaluation. Top-*k* represents the mean of the *k* highest ECFP6 Tanimoto similarities between generated molecules and known inhibitors of the corresponding target. Values are reported as mean ± standard deviation across five generation seeds. Bold indicates the higher mean for each target and metric; ties are bolded for both models.

As in the KD analysis, molecules generated by DEG2MOL overlapped more closely with the reference property distributions (Supporting Information, Figure S5). Following target-specific reference molecular-weight matching, DEG2MOL achieved higher target-averaged top-1, top-10, and top-100 similarities (0.758 vs 0.531, *q* = 0.016, 0.611 vs 0.429, *q* = 0.009, and 0.385 vs 0.305, *q* = 0.009, respectively). DEG2MOL was higher for all seven targets at top-10 and top-100 and for six of the seven targets at top-1, with HDAC1 as the only top-1 exception. We also visualized the molecules generated for each target gene (Figure 6). The visualization confirms that DEG2MOL generated molecular structures that closely recapitulated the principal scaffolds of known inhibitors.

**Figure 6:**
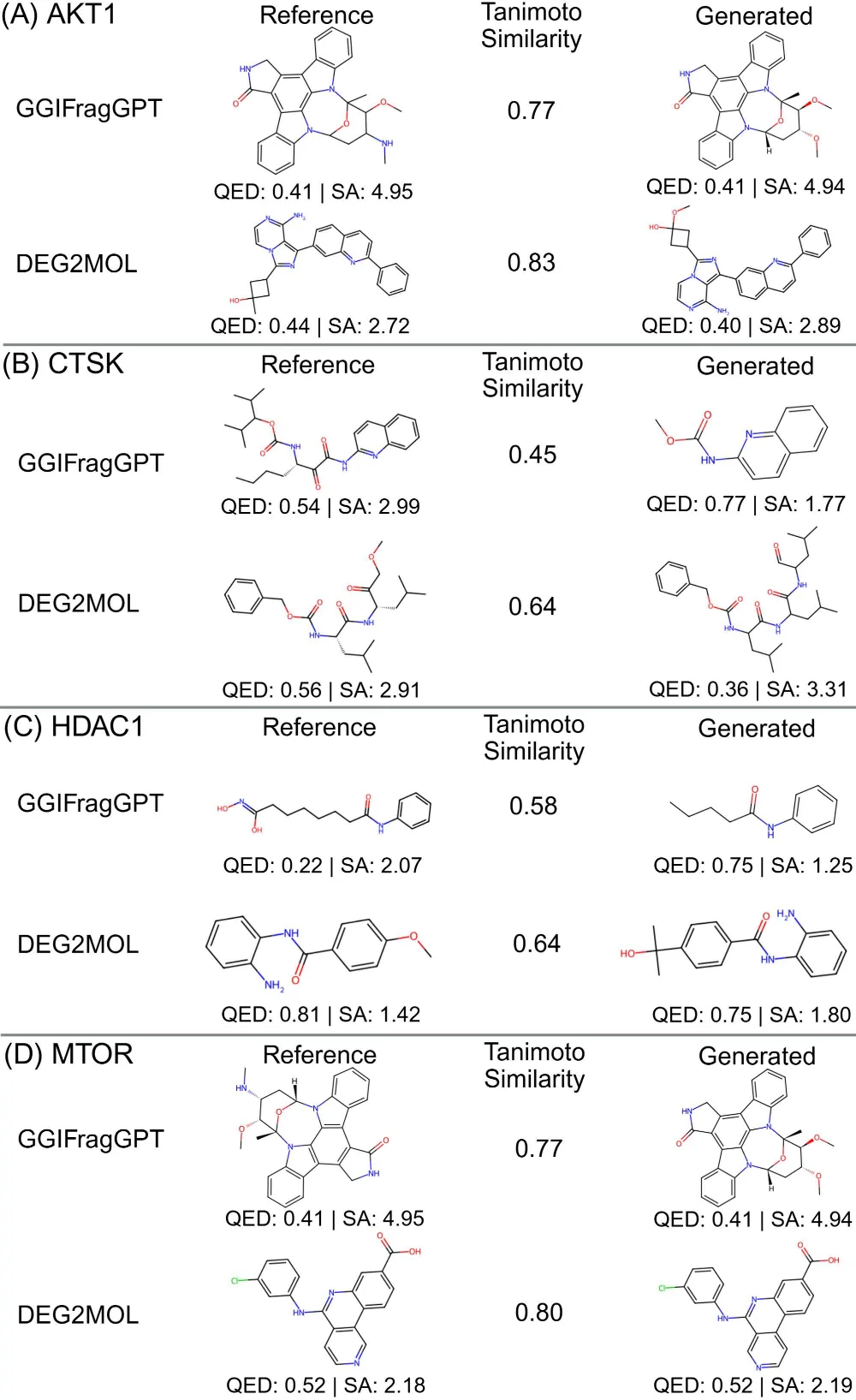
Visualization of molecules generated from LINCS L1000 knockout-induced DEG profiles. For each therapeutic target, (A) AKT1, (B) CTSK, (C) HDAC1, (D) MTOR, both GGIFragGPT (top) and DEG2MOL (bottom) generated 100 molecules. For each panel, the molecule that achieved the maximum ECFP6 Tanimoto similarity to the known reference inhibitor curated from ExCAPE is shown. QED and SA scores are labeled for both reference and generated molecules.

Across the KO targets, the target-averaged top-*k* similarities were lower than in the KD setting (top-1:0.800 vs 0.916; top-10:0.635 vs 0.810; top-100:0.410 vs 0.536). This may reflect differences in perturbation modality; compound-induced transcriptomic profiles more closely resemble KD, as both represent partial suppression of target activity, whereas KO induces complete ablation of gene function.^64^ Such differences in perturbation magnitude may produce transcriptomic signatures that are less directly comparable to the compound-induced profiles. DEG2MOL nonetheless obtained higher top-1 similarities for six of the seven KO targets without additional training.

### Molecular Generation from Perturb-seq DEG Profiles

To evaluate whether DEG2MOL transfers to a different transcriptomic profiling platform, we generated molecules from Perturb-seq CRISPRi profiles^65^ by aggregating single-cell profiles into pseudobulk profiles using decoupler,^66^ following the LINCS L1000 Level 5 normalization protocol. For feature alignment, we retained genes that overlapped with the LINCS L1000 dataset and imputed zero values for any unmatched genes. Because the training data were microarray-derived whereas the Perturb-seq profiles were single-cell RNA-seq-derived, we first assessed whether the two platforms were comparable. A permutation test based on Euclidean distances indicated that Perturb-seq and LINCS L1000 KD profiles matched by target gene and cell line were closer than mismatched controls (*p* = 2.54 × 10^–5^). An additional source-mixing analysis further showed that LINCS L1000 shRNA and Perturb-seq CRISPRi DEG profiles were not separated by data source across all available samples (Supporting Information, Figure S6).

We visualized the molecules generated for AKT1, MTOR, and PIK3CA to evaluate their structural and chemical properties compared to known inhibitors of the corresponding target (Figure 7A–C). Representative structures recovered structural similarity to known inhibitors, suggesting that Perturb-seq profiles provide informative signals for molecular generation and supporting the potential applicability of the model across different transcriptomic profiling platforms.

**Figure 7:**
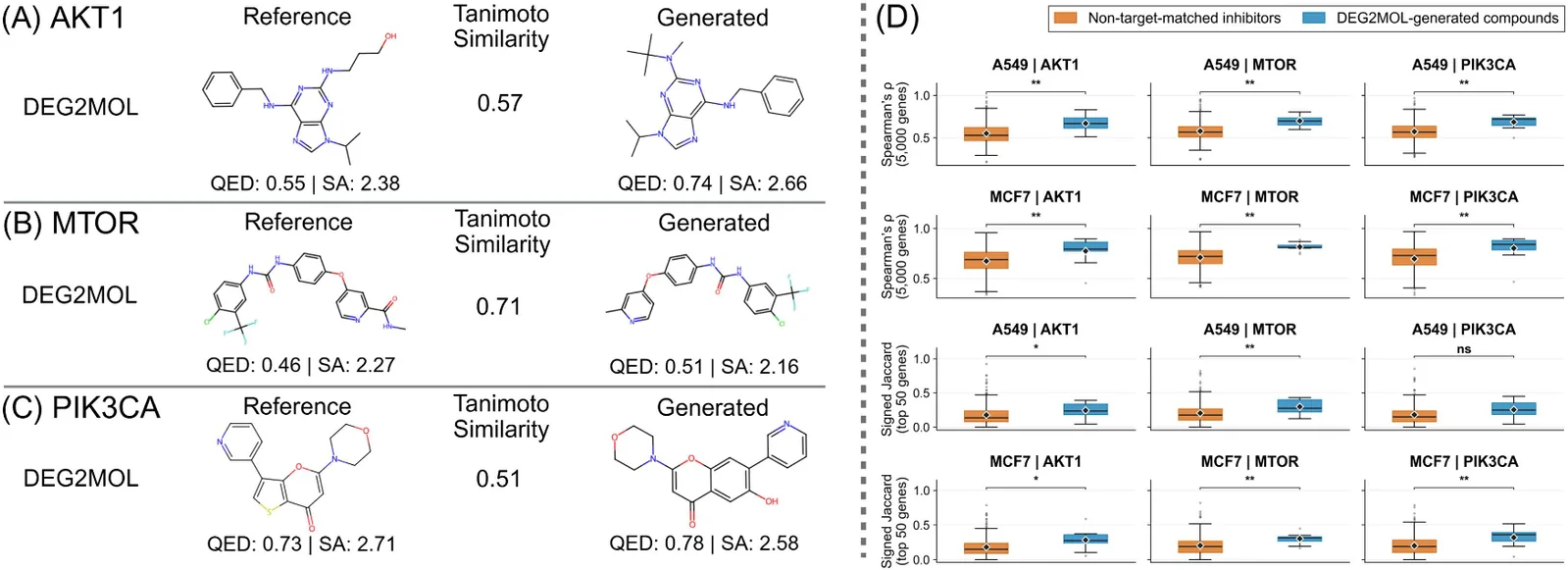
Visualization of molecules generated by DEG2MOL from Perturb-seq DEG profiles and comparison using CRISP-predicted transcriptional profiles. (A–C) Representative examples of molecules generated by the model conditioned on Perturb-seq profiles targeting AKT1, MTOR, and PIK3CA, respectively. Each panel displays the known reference inhibitor (left) alongside the generated molecule (right) that achieved the maximum ECFP6 Tanimoto similarity. Tanimoto similarity, QED, and SA scores are labeled for both the reference and generated structures. (D) Comparison of CRISP-predicted transcriptional profile similarity between target-matched reference molecules and either the corresponding profile of DEG2MOL-generated molecules or non-target-matched comparator molecules. Target-matched reference profiles were compared with the corresponding cell- and target-matched profile of DEG2MOL-generated molecules (blue) or with profiles of comparator molecules targeting AURKB, HDAC1, and CTSK (orange). Similarity was measured using Spearman’s *ρ* across 5000 genes (top two rows) and Top-50 signed Jaccard similarity (bottom two rows). Boxes indicate the interquartile range, center lines indicate medians, diamonds indicate means, and whiskers extend to 1.5× the interquartile range. *q < 0.05, **q < 0.01, and ***q < 0.001; ns, *q* ≥ 0.05.

Structural similarity to known inhibitors, however, does not indicate whether generated molecules would induce related transcriptional responses, and to our knowledge, this aspect has rarely been evaluated in existing transcriptome-guided generative approaches. We therefore used CRISP to predict compound-induced gene expression changes *in silico*. We evaluated whether the predicted transcriptional profiles of DEG2MOL-generated molecules were more similar to target-matched reference molecules than to non-target-matched comparator molecules (Figure 7D). Across all six cell line–target combinations, target-matched reference profiles showed greater mean similarity to the corresponding representative generated-molecule profiles than to the non-target-matched comparator profiles. Mean Spearman correlations ranged from 0.669 to 0.814 for the reference–generated-profile comparisons and from 0.551 to 0.709 for the reference–comparator comparisons. The mean paired differences in similarity were positive in all six combinations and ranged from 0.101 to 0.120, with all six comparisons remaining significant (*q* ≤ 0.00335). Mean Top-50 signed Jaccard similarities ranged from 0.241 to 0.319 for the reference-generated-profile comparisons and from 0.177 to 0.203 for the reference–comparator comparisons. The mean paired differences were positive in all six cell line–target combinations and ranged from 0.064 to 0.118. Five of the six comparisons remained significant after correction, whereas A549-PIK3CA did not reach the corrected significance threshold (*q* = 0.0527). These results provide complementary *in silico* support that the aggregate predicted profiles of DEG2MOL-generated molecules are modestly more similar to target-matched reference molecules than to the selected non-target-matched comparators. Because all compound-induced profiles in this analysis were computationally predicted by CRISP, these findings should be interpreted as a supplementary assessment of predicted transcriptional similarity rather than as functional validation.

## Discussion

Several key design choices of DEG2MOL enable the breadth of generalization. By conditioning generation on DEG profiles rather than post-treatment expression values, DEG2MOL isolates compound-specific transcriptional signals from cell line-specific confounders, providing a cleaner biological condition for molecular generation. The GO-informed DEG encoder further incorporates hierarchical biological knowledge into the transcriptomic representation, so that individual latent dimensions can be annotated with their associated GO terms. These analyses characterize the structure of the latent space descriptively and do not establish mechanistic drug action (Supporting Information, Figures S7 and S8). Beyond this latent-space characterization, the GO–pharmacophore analysis of representative compound conditions provided condition-level evidence linking GO-aligned node activations to the prevalence of relevant pharmacophore features in the generated molecules. Classifier-free guidance further strengthens the alignment of generated structures to the given condition.^67^ The ablation study confirmed that removing either the GO-BP hierarchy or DEG conditioning led to a consistent decrease in structural similarity across all metrics (Supporting Information, Figure S9). Together, these components support condition-aligned molecular generation in the diverse perturbation settings evaluated.

Despite these strengths, our model has several limitations. First, the reliance on a frozen, pre-trained scaffold-aware VAE backbone and a separately trained DEG encoder constrains the framework to a fixed molecular latent space, limiting its capacity for end-to-end adaptation. Second, the number of available DEG profile-molecule pairs remains relatively small, which may limit the model’s exposure to diverse chemical and transcriptomic spaces. Third, the GO-aligned node activations support condition-level association rather than causal attribution; elevated activation may reflect indirect downstream or compensatory transcriptional responses, so the encoder is described as GO-annotatable rather than mechanistically explanatory. Finally, neither the generated molecules nor the CRISP-based transcriptomic predictions have been experimentally validated; therefore, the docking and transcriptomic-response analyses should be interpreted as supportive *in silico* evidence rather than direct validation of binding affinity, target engagement, cellular activity, or therapeutic potential.

Several potential failure scenarios were also noted. A fundamental limitation of DEG2MOL is its reliance on transcriptional perturbation signals as the only conditioning input. For biological perturbations that operate primarily at the protein level without inducing substantial transcriptional changes, such as protein truncation mutations or post-translational modifications, DEG profiles may provide insufficient conditioning signals, limiting the applicability of the framework to such contexts. Moreover, because the molecular latent space is defined by a pretrained ScafVAE, structurally distinct chemotypes outside the chemical space represented in ChEMBL and ZINC may remain inaccessible.

Future work will prioritize the experimental validation of the generated molecules and elucidation of their specific biological targets. We also aim to develop an end-to-end generative framework that jointly optimizes transcriptomic encoding and molecular generation and to expand the training dataset with more diverse DEG profile-molecule pairs. These advances will strengthen the biological grounding of transcriptome-guided *de novo* drug design and bring the framework closer to practical applications in real-world drug discovery pipelines.

DEG2MOL was developed as a biologically grounded conditioning framework for transcriptome-guided *de novo* drug design. Across the compound-induced evaluations, the model achieved the highest rank-sum performance under both random and scaffold splits, and the latent-space, GO–pharmacophore, and docking analyses consistently showed condition-associated patterns. The application of the same trained model to KD-induced, KO-induced, and Perturb-seq profiles further extended the evaluation beyond the compound perturbations used for training. Together, these results characterize the framework across multiple perturbation settings while retaining a common DEG-based conditioning strategy.

## Conclusion

DEG2MOL integrates a GO-informed DEG encoder with a pre-trained scaffold-aware VAE through conditional latent FM for transcriptome-guided *de novo* drug design. In the compound-induced evaluations, the framework ranked first by rank-sum aggregation under both random and scaffold splits. The GO–pharmacophore and docking analyses further showed condition-associated biological and chemical features in representative cases. Without additional training, DEG2MOL also generated molecules from KD-induced, KO-induced, and Perturb-seq profiles that showed target-related structural similarity. For the Perturb-seq conditions, CRISP-based analysis provided complementary evidence of predicted transcriptional similarity to target-matched reference compounds. These results support condition-aligned molecular generation across the perturbation settings and profiling platforms evaluated, while the docking and predicted transcriptomic analyses remain *in silico* assessments requiring experimental validation.

## Supplementary Material



## Data Availability

The data and code are available at https://github.com/KU-MedAI/DEG2MOL.

## Notes

DEG Autoencoder Architecture; Hyperparameter Search Space for the DEG Encoder; Per-Metric Ranks Underlying the Aggregate Model Ranking; Projection of Test-Set Compound Representations onto the Train Manifold; Reference Molecular-Weight-Matched Analysis of KD- and KO-Conditioned Molecules; Assessment of Batch Effects between L1000 shRNA and Perturb-seq CRISPRi Profiles; Analysis of DEG Latent Vectors; Interpretation of DEG Encoder; Ablation Study (PDF)
