# Supplementary material for: DEG2MOL: Conditional Latent Flow Matching for Transcriptome-Guided de novo Drug Design
Source: ACS Omega. 2026 Sep 28;11(40):61631–45. doi: 10.1021/acsomega.6c07302 (PMC13670877; doi:10.1021/acsomega.6c07302)
Supplement: Supplementary file 1 [file ao6c07302_si_001.pdf]

Supporting Information

DEG2MOL: Conditional Latent Flow Matching for Transcriptome-Guided *De Novo* Drug Design

Songhyeon Kim,[1,†] Wootaek Lim,[1,†] Hawon Lee,[1] Hyunsu Bong,[1] Jitae Kim[1] and Minji Jeon [1,2,*]

[1]Department of Biomedical Sciences, Korea University College of Medicine, 73 Goryeodae-ro, 02841, Seoul, Republic of Korea

[2]Department of Biomedical Informatics, Korea University College of Medicine, 161 Jeongreung-ro, 02708, Seoul, Republic of Korea

[†]Equal contribution. [*]Corresponding author. mjjeon@korea.ac.kr

S1. DEG Autoencoder Architecture

The differentially expressed genes (DEG) autoencoder (AE) consists of an encoder E_{deg} and a decoder D_{deg} with symmetric architectures that compress and reconstruct high-dimensional DEG profiles $g \in R^{10280}$ through a compact latent vector $c \in R^{64}$. The architecture mirrors the Gene Ontology (GO) [1] hierarchical structure to incorporate biological pathway priors, with encoder layer sizes of 2,011, 1,614, and 1,075 nodes, corresponding to GO levels 7, 6, and 5, respectively. Each layer l performs the following operation:

$$h^{(l)} = \mathrm{Mish}\left(\mathrm{BN}\left(W^{(l)}h^{(l-1)} + b^{(l)}\right)\right) \quad (1)$$

where $W^{(l)}$ and $b^{(l)}$ represent the weight matrix and bias vector, respectively. BN denotes batch normalization and $\mathrm{Mish}(x) = x \cdot \tanh(\mathrm{softplus}(x))$ is the activation function. To ensure accurate reconstruction of DEG profiles while preserving biologically relevant structure, the model is trained by minimizing a multi-component loss function as follows:

$$L_{total} = L_{rec} + \alpha L_{PCC} + \lambda L_{mask} \quad (2)$$

where α and λ are hyperparameters that control the contributions of the L_{PCC} and L_{mask} and are set to 5 and 0.00001, respectively. The first term, L_{rec}, represents the reconstruction loss calculated as the mean absolute error (MAE) between the input profile g and the reconstructed profile $\hat{g}$ as follows:

$$L_{rec} = \frac{1}{N \cdot D} i = 1N \sum_{j=1}^{D} \left|g_{i,j} - \widehat{g_{i,j}}\right| \quad (3)$$

where N denotes the batch size and D represents the dimensionality of the gene expression profile (i.e., the number of genes). The indices i and j refer to the i-th sample in the batch and the j-th gene feature, respectively.

The second term, L_{PCC}, is the Pearson correlation coefficient (PCC) loss, which preserves the relative expression patterns across profiles by minimizing the term 1 – PCC between the original and reconstructed vectors as follows:

$$L_{PCC} = \frac{1}{N}\sum_{i=1}^{N}\left(1 - \frac{\sum_{j=1}^{D}(g_{i,j} - \mu_{x_i})(\widehat{g_{i,j}} - \mu_{\widehat{g_i}})}{\sqrt{\sum_{j=1}^{D}(g_{i,j} - \mu_{g_i})^2 + \epsilon} \cdot \sqrt{\sum_{j=1}^{D}(\widehat{g_{i,j}} - \mu_{\widehat{g_i}})^2 + \epsilon}}\right) \quad (4)$$

Here, μ_{g_i} and $\mu_{\hat{g}_i}$ denote the mean expression values of the *i*-th original and reconstructed profiles, respectively. A small constant ϵ (e.g., 10^{-8}) is added to the denominator to prevent numerical instability during division.

In addition, the third term, L_{mask}, embeds prior biological knowledge by selectively penalizing weights between nodes that do not have real biological relationships as specified by the GO hierarchy, as follows:

$$L_{mask} = \sum_{l=1}^{L-1} |W^{(l)} \odot (1 - C^{(l)})|_1 \quad (5)$$

where L denotes the total number of layers in the hierarchy-informed encoder and $W^{(l)}$ represents the weight matrix of the *l*-th layer. The operator $\odot$ denotes the element-wise (Hadamard) product. $C^{(l)}$ is a predefined binary connectivity matrix derived from the GO connection network data, where an entry is set to 1 if a biological connection exists between nodes (genes or GO terms), and 0 otherwise. Consequently, the term (1 - $C^{(l)}$) acts as an inverse mask that activates penalties only for weights corresponding to non-existent biological links, suppressing them via the L1-norm regularization denoted by $\|\cdot\|_1$.

S2. Hyperparameter search space for DEG encoder

To identify the optimal architectural configuration for the DEG encoder, we conducted a systematic hyperparameter search across GO ontology categories, level combinations, optimizer, activation function, and regularization terms (Table S1). Based on this search, the GO-Biological Process (BP) with levels 7, 6, and 5 were selected as the optimal architectural configuration, yielding sequential layers with 2,011, 1,614, and 1,075 nodes, respectively. The final model was trained using the AdamW optimizer with the Mish activation function, and the L2 regularization term of 0.00001.

Table S1 Hyperparameter search space

Parameter	Search space	Selected hyperparameter
**GO**	Biological Process (BP), Cellular Component (CC), Molecular Function (MF)	BP
**GO level**	Any combination of two or more consecutive levels between [1, 5]	[7, 6, 5]
**Optimizer**	Adam, AdamW	AdamW
**Activation function**	{ReLU, tanh, Leaky ReLU, Mish}	Mish
**L2 regularization term**	{0, 0.00001, 0.0001, 0.001, 0.01, 0.1}	0.00001

S3. Per-Metric Ranks Underlying the Aggregate Model Ranking

The Rank column in main-text Table 1 summarizes model performance across six evaluation metrics: validity, uniqueness, novelty, ECFP6 similarity, MACCS similarity, and Fraggle similarity. For each metric and data split, the metric values were first averaged across the five generation seeds (0, 1, 2, 3, and 42), and models were ranked from best to worst. Ties were assigned midranks.

The six per-metric ranks were summed within each split, with lower rank-sums indicating better aggregate performance. The corresponding mean rank was calculated by dividing the rank-sum by six, and the split-level ordinal rank reported in Table 1 was determined from the rank-sum. Table S2 reports the complete per-metric ranks and split-level aggregates underlying the Rank column in Table 1.

Table S2 Per-metric ranks and rank-sum aggregation for the random and scaffold splits. Metric values were averaged across five generation seeds before ranking. Rank 1 indicates the best performance for each metric. Per-metric ties were assigned midranks. Rank Sum is the sum of the six metric-specific ranks, and Mean Rank is Rank Sum divided by six. Split Rank orders models within each split according to Rank Sum. The across-split overall rank was obtained by summing the random- and scaffold-split rank-sums with equal weight. Internal diversity and scaffold diversity were excluded from the rank-sum analysis.

Split	Model	Val Rank	Uniq Rank	Novel Rank	ECFP6 Rank	MACCS Rank	Fraggle Rank	Rank Sum	Mean Rank	Rank
Random	TransGEM	1.5	6	5	6	6	6	30.5	5.08	6
	FAME	6	2	2	5	5	5	25.0	4.17	5
	GxVAEs	5	3	6	2	2	2	20.0	3.33	3
	BiCEV	4	5	1	4	4	4	22.0	3.67	4
	GGIFragGPT	1.5	4	3	3	3	3	17.5	2.92	2
	DEG2MOL	3	1	4	1	1	1	11.0	1.83	1
Scaffold	TransGEM	1	6	1.5	6	6	6	26.5	4.42	5
	FAME	5	3	3	4	4	4	23.0	3.83	4
	GxVAEs	6	1	6	5	5	5	28.0	4.67	6
	BiCEV	4	5	1.5	3	3	3	19.5	3.25	3
	GGIFragGPT	2	4	4	2	2	2	16.0	2.67	2
	DEG2MOL	3	2	5	1	1	1	13.0	2.17	1

S4. Projection of Test-Set Compound Representations onto the Train Manifold

To assess whether the compound-specificity of molecular latent vectors extends beyond pairwise similarity to the broader structure of the latent space, we performed a UMAP projection of all molecular latent vectors generated from compound-induced DEG profiles in both the training and test sets. Specifically, the UMAP embedding was fit on the train-set latent vectors, and the test-set latent vectors were subsequently projected onto this fixed embedding without refitting, thereby providing an unbiased view of how unseen compound profiles are situated relative to the learned train manifold.

For the 20 most-represented compounds shared between the training and test sets, we computed the centroid of each compound's latent vectors separately within the training split (circle markers) and the test split (triangle markers), and connected each pair of split centroids with a line segment to facilitate visual comparison of their relative positions (Figure S1). Across nearly all compounds, the training and test centroids co-localized within the same region of the embedding, with the connecting line segments remaining notably short relative to the overall extent of the latent space. This spatial congruence indicates that DEG2MOL consistently maps the transcriptomic signatures of a given compound to the same latent neighborhood, irrespective of whether those signatures were encountered during training or withheld for evaluation. Taken together, these results confirm that the compound-specific clustering observed in pairwise cosine similarity analyses (Figure 2) reflects a systematic and globally coherent organizational principle of the learned latent space, rather than a local or incidental pattern.

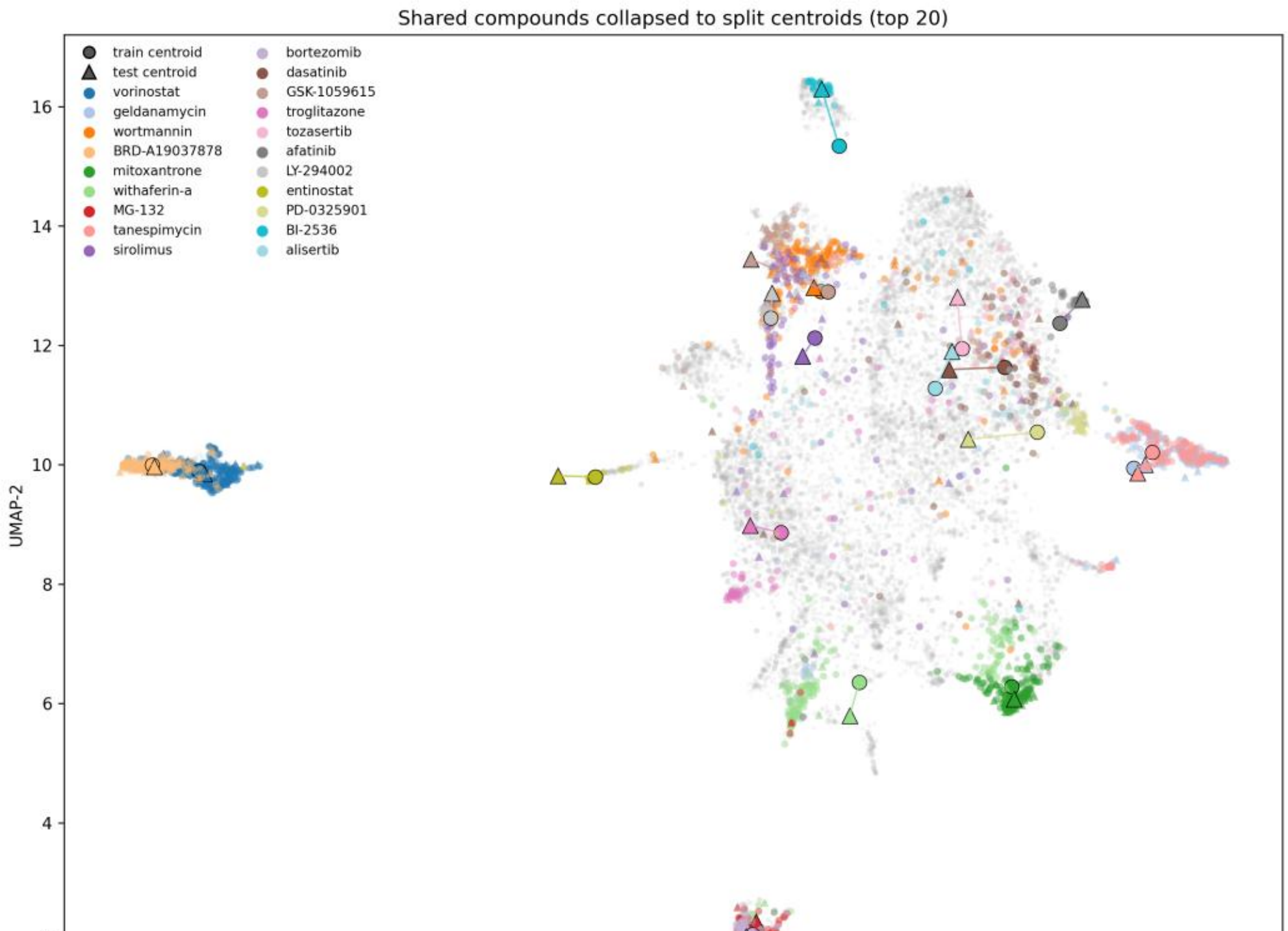

Figure S1 UMAP projection of molecular latent vectors generated from compound-induced DEG profiles, illustrating the co-localization of training- and test-set representations for shared compounds. Each point represents a single molecular latent vector; colors denote compound identity, and gray points correspond to compounds not among the top 20 most-represented shared compounds. Circle markers (train centroid) and triangle markers (test centroid) indicate the centroids of the training- and test-set latent vectors, respectively, for each of the 20 shared compounds with the highest number of DEG profiles. Line segments connect the training and test centroids of the same compound to facilitate direct visual comparison of their relative positions in the embedding. The UMAP embedding was fit exclusively on train-set latent vectors; test-set vectors were projected onto the fixed embedding without refitting.

S5. Reference Molecular-Weight-Matched Analysis of KD- and KO-Conditioned Molecules

The unfiltered KD and KO analyses showed that DEG2MOL generally achieved higher Top-1 and Top-10 ECFP6 similarities, whereas GGIFragGPT was higher for several targets at Top-100. To examine whether these target-level differences were associated with the physicochemical ranges sampled by the two models, we compared the property distributions of the generated molecules with those of the corresponding reference inhibitors. Molecules generated by DEG2MOL showed greater overlap with the reference distributions of molecular weight (MW), logP, QED, TPSA, and heavy-atom count in both the KD and KO settings (Figure S2).

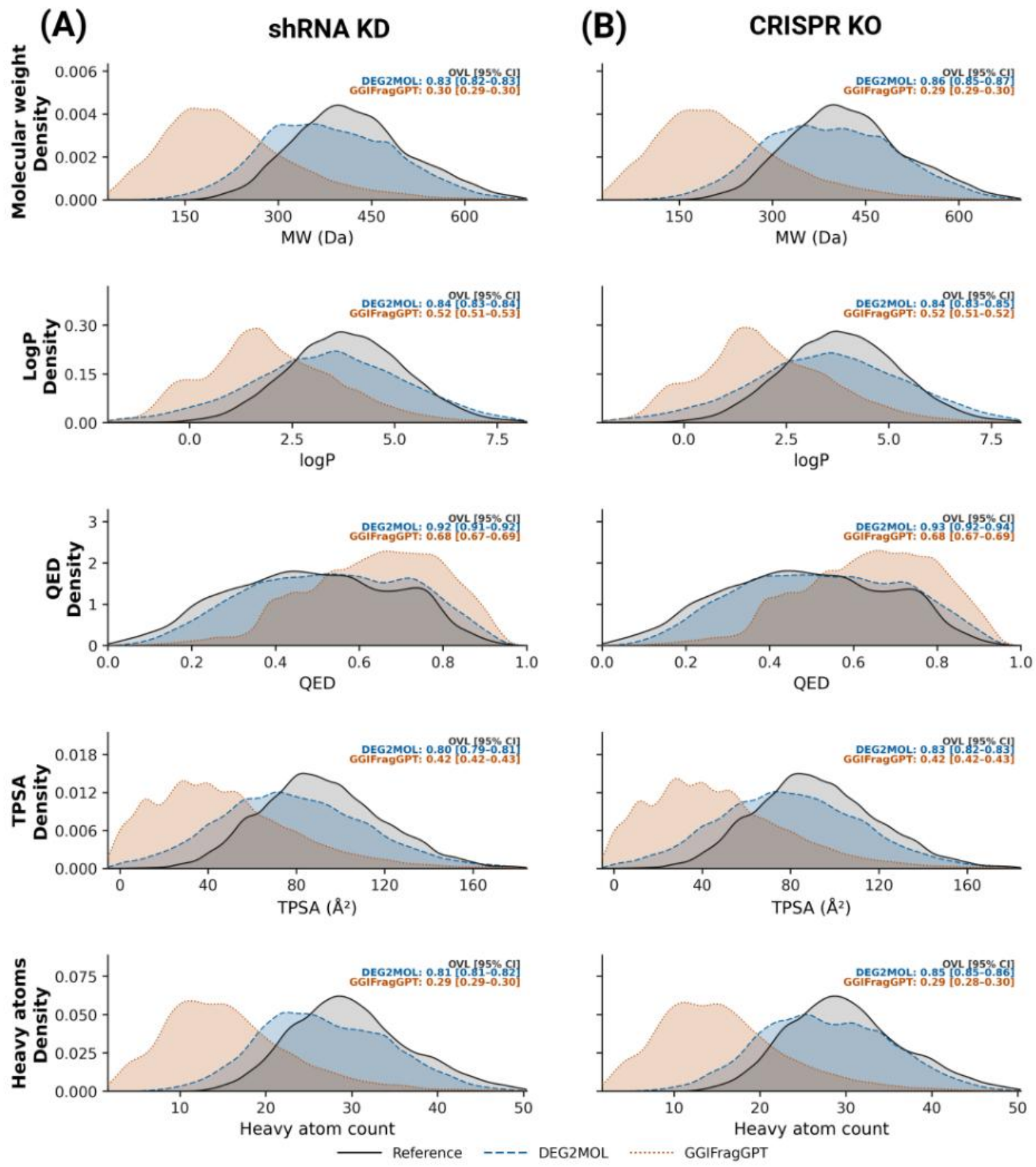

Figure S2 Physicochemical-property distributions of reference and generated molecules in the (A) shRNA KD and (B) CRISPR KO evaluations. Kernel density estimates are shown for molecular weight (MW), logP, quantitative estimate of drug-likeness (QED), topological polar surface area (TPSA), and heavy-atom count. Black solid lines represent the target-associated reference inhibitors, blue dashed lines represent molecules generated by DEG2MOL, and orange dotted lines represent molecules generated by GGIFragGPT. The overlap coefficient (OVL) between each generated-molecule distribution and the corresponding reference distribution is reported in each panel with its 95% confidence interval. Higher OVL values indicate greater distributional overlap with the reference molecules.

We therefore performed a reference MW-matched sensitivity analysis to compare the models' ability to generate molecules structurally similar to known target inhibitors within a comparable, reference-like MW range. This analysis was not intended to evaluate generation performance as a function of MW; rather, it tested whether the ECFP6 similarity patterns observed in the unfiltered candidate pools persisted after reducing between-model differences in MW distribution. Each model generated 100 molecules per input DEG profile and generation seed. Molecules were pooled by target and retained when their MW fell within the mean ± one standard deviation of the corresponding reference-inhibitor

distribution. For each retained molecule, we calculated the maximum ECFP6 Tanimoto similarity to the target's reference inhibitors and summarized the highest 1, 10, and 100 scores. Pass count was reported only to document the number of candidates remaining after MW matching, whereas the Top-k similarities were used to evaluate structural proximity to the reference inhibitors. All values are reported as mean ± standard deviation across generation seeds.

As shown in Table S3, in the shRNA KD evaluation, DEG2MOL retained more candidates within the reference MW window than GGIFragGPT for all eight targets and achieved higher Top-1, Top-10, and Top-100 similarities for every target. Averaged across targets, the Top-1, Top-10, and Top-100 similarities were 0.881, 0.777, and 0.504 for DEG2MOL, compared with 0.634, 0.529, and 0.402 for GGIFragGPT, respectively. In contrast to the unfiltered Top-100 analysis, in which GGIFragGPT was higher for EGFR, HDAC1, and PIK3CA, DEG2MOL was higher for all eight targets after MW matching. Thus, the stronger structural similarity of the KD-conditioned molecules generated by DEG2MOL persisted when the comparison was restricted to candidates within the reference-like MW range.

Table S3 Reference molecular-weight-matched analysis for molecules generated from shRNA knockdown-induced DEG profiles. 100 molecules were generated independently for each input DEG profile, and molecules from profiles associated with the same target were pooled within each generation run before applying the molecular-weight filter. The molecular-weight window for each target was defined as the mean and one standard deviation of the corresponding reference inhibitors. Pass count and Top-k ECFP6 Tanimoto similarities are reported as mean and standard deviation across generation seeds.

Target Gene	MW Window	Model	Pass Count	Top-1	Top-10	Top-100
**AKT1**	323.4-522.7	DEG2MOL	15,144 ± 1,160	1.000 ± 0.000	0.930 ± 0.108	0.622 ± 0.099
	323.4-522.7	GGIFragGPT	3,731 ± 378	0.714 ± 0.058	0.619 ± 0.076	0.458 ± 0.077
**AKT2**	303.7-511.6	DEG2MOL	9,823 ± 578	1.000 ± 0.000	0.823 ± 0.059	0.489 ± 0.052
	303.7-511.6	GGIFragGPT	2,801 ± 281	0.612 ± 0.105	0.534 ± 0.119	0.401 ± 0.036
**AURKB**	317.8-508.9	DEG2MOL	7,994 ± 449	1.000 ± 0.000	0.815 ± 0.110	0.485 ± 0.059
	317.8-508.9	GGIFragGPT	2,172 ± 256	0.669 ± 0.164	0.556 ± 0.120	0.407 ± 0.049
**CTSK**	320.3-545.2	DEG2MOL	1,961 ± 228	0.681 ± 0.033	0.641 ± 0.036	0.402 ± 0.053
	320.3-545.2	GGIFragGPT	474 ± 79	0.372 ± 0.020	0.331 ± 0.003	0.270 ± 0.004
**EGFR**	321.9-520.7	DEG2MOL	19,818 ± 1,608	1.000 ± 0.000	0.961 ± 0.050	0.633 ± 0.077
	321.9-520.7	GGIFragGPT	4,936 ± 511	0.837 ± 0.182	0.655 ± 0.081	0.533 ± 0.059
**HDAC1**	318.8-525.4	DEG2MOL	9,573 ± 756	0.608 ± 0.075	0.500 ± 0.036	0.404 ± 0.009
	318.8-525.4	GGIFragGPT	2,366 ± 121	0.521 ± 0.022	0.463 ± 0.014	0.372 ± 0.008
**MTOR**	342.0-521.6	DEG2MOL	9,536 ± 952	1.000 ± 0.000	0.976 ± 0.041	0.599 ± 0.046
	342.0-521.6	GGIFragGPT	2,154 ± 150	0.699 ± 0.072	0.553 ± 0.106	0.405 ± 0.054
**PIK3CA**	335.2-513.0	DEG2MOL	7,880 ± 590	0.758 ± 0.195	0.573 ± 0.080	0.394 ± 0.029
	335.2-513.0	GGIFragGPT	1,748 ± 179	0.644 ± 0.188	0.524 ± 0.137	0.371 ± 0.051

As shown in Table S4, in the CRISPR KO evaluation, DEG2MOL retained more candidates within the reference MW window than GGIFragGPT for all seven targets. DEG2MOL also achieved higher Top-10 and Top-100 similarities for every target and higher Top-1 similarity for six of the seven targets. HDAC1 was the only Top-1 exception, where GGIFragGPT was marginally higher than DEG2MOL (0.444 vs. 0.437); however, DEG2MOL remained higher for HDAC1 at Top-10 (0.396 vs. 0.372) and Top-100 (0.323 vs. 0.284). Averaged across targets, the Top-1, Top-10, and Top-100 similarities were 0.758, 0.611, and 0.385 for DEG2MOL, compared with 0.531, 0.429, and 0.305 for GGIFragGPT, respectively. Notably, the mixed Top-100 pattern observed in the unfiltered KO candidate pools did not persist after MW matching: DEG2MOL was higher for all seven targets and at the target-averaged level. These results show that DEG2MOL more consistently generated KO-conditioned molecules structurally similar to known inhibitors when the candidates were compared within a comparable MW range.

Table S4 Reference molecular-weight-matched analysis for molecules generated from CRISPR knockout-induced DEG profiles. 100 molecules were generated independently for each input DEG profile, and molecules from profiles associated with the same target were pooled within each generation run before applying the molecular-weight filter. The molecular-weight window for each target was defined as the mean and one standard deviation of the corresponding reference inhibitors. Pass count and Top-k ECFP6 Tanimoto similarities are reported as mean and standard deviation across generation seeds.

Target Gene	MW Window	Model	Pass Count	Top-1	Top-10	Top-100
**AKT1**	323.4-522.7	DEG2MOL	6,759 ± 349	1.000 ± 0.000	0.929 ± 0.045	0.519 ± 0.035
	323.4-522.7	GGIFragGPT	1,466 ± 193	0.706 ± 0.098	0.582 ± 0.068	0.384 ± 0.016
**AURKB**	317.8-508.9	DEG2MOL	412 ± 16	0.603 ± 0.206	0.428 ± 0.040	0.287 ± 0.012
	317.8-508.9	GGIFragGPT	89 ± 13	0.438 ± 0.055	0.355 ± 0.018	0.246 ± 0.006
**CTSK**	320.3-545.2	DEG2MOL	1,342 ± 70	0.677 ± 0.049	0.613 ± 0.056	0.354 ± 0.036
	320.3-545.2	GGIFragGPT	286 ± 22	0.399 ± 0.025	0.334 ± 0.009	0.258 ± 0.008
**EGFR**	321.9-520.7	DEG2MOL	4,352 ± 166	1.000 ± 0.000	0.762 ± 0.057	0.463 ± 0.038
	321.9-520.7	GGIFragGPT	980 ± 99	0.686 ± 0.053	0.583 ± 0.067	0.399 ± 0.011
**HDAC1**	318.8-525.4	DEG2MOL	1,600 ± 81	0.437 ± 0.028	0.396 ± 0.005	0.323 ± 0.009
	318.8-525.4	GGIFragGPT	357 ± 25	0.444 ± 0.049	0.372 ± 0.015	0.284 ± 0.005
**MTOR**	342.0-521.6	DEG2MOL	1,733 ± 83	0.953 ± 0.114	0.688 ± 0.088	0.439 ± 0.038
	342.0-521.6	GGIFragGPT	319 ± 55	0.568 ± 0.119	0.418 ± 0.046	0.306 ± 0.006
**PIK3CA**	335.2-513.0	DEG2MOL	1,022 ± 69	0.635 ± 0.078	0.460 ± 0.034	0.307 ± 0.015
	335.2-513.0	GGIFragGPT	208 ± 48	0.475 ± 0.060	0.356 ± 0.018	0.260 ± 0.012

S6. Assessment of Batch Effects between L1000 shRNA and Perturb-seq CRISPRi Profiles

We compared DEG profiles from LINCS L1000 shRNA and Perturb-seq CRISPRi to evaluate whether the profiles were separated by data source. UMAP visualization was performed using all available profiles. Source mixing was quantified using the scIB [2] batch average silhouette width (ASW), with data source used as the batch label. This score ranges from 0 to 1, where higher values indicate better mixing between sources. The DEG profiles showed a high batch ASW of 0.9646 in the all-sample comparison. These results indicate that the DEG profiles did not show source-driven separation between LINCS L1000 shRNA and Perturb-seq CRISPRi, supporting their use in cross-source perturbation analysis.

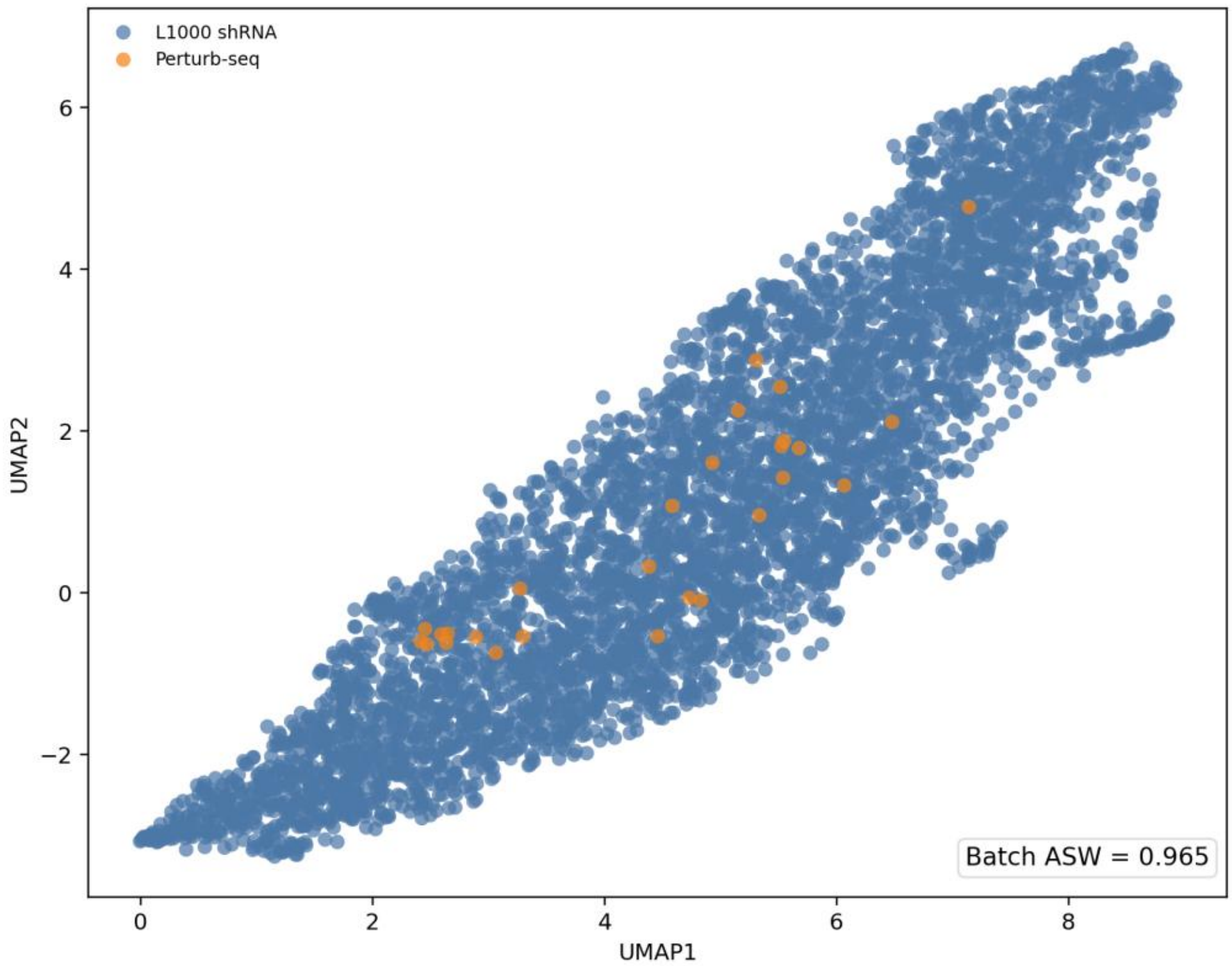

Figure S3 UMAP visualization of DEG profiles from LINCS L1000 shRNA and Perturb-seq CRISPRi using all available profiles. Points are colored by data source. The profiles did not show strong source-driven separation, consistent with the high batch ASW score of 0.9646.

S7. Analysis of DEG Latent Vectors

To verify that the DEG encoder produces condition-specific latent vectors, we computed pairwise cosine similarities among latent vectors generated from compound-induced DEG profiles in the test set. We compared similarities between latent vectors derived from the same compound across different cell lines with those between vectors derived from distinct compounds (Figure S4). Latent vectors from the same compound showed significantly higher cosine similarity than those from different compounds, indicating that the representation captures compound-specific transcriptional patterns despite variation in cellular context.

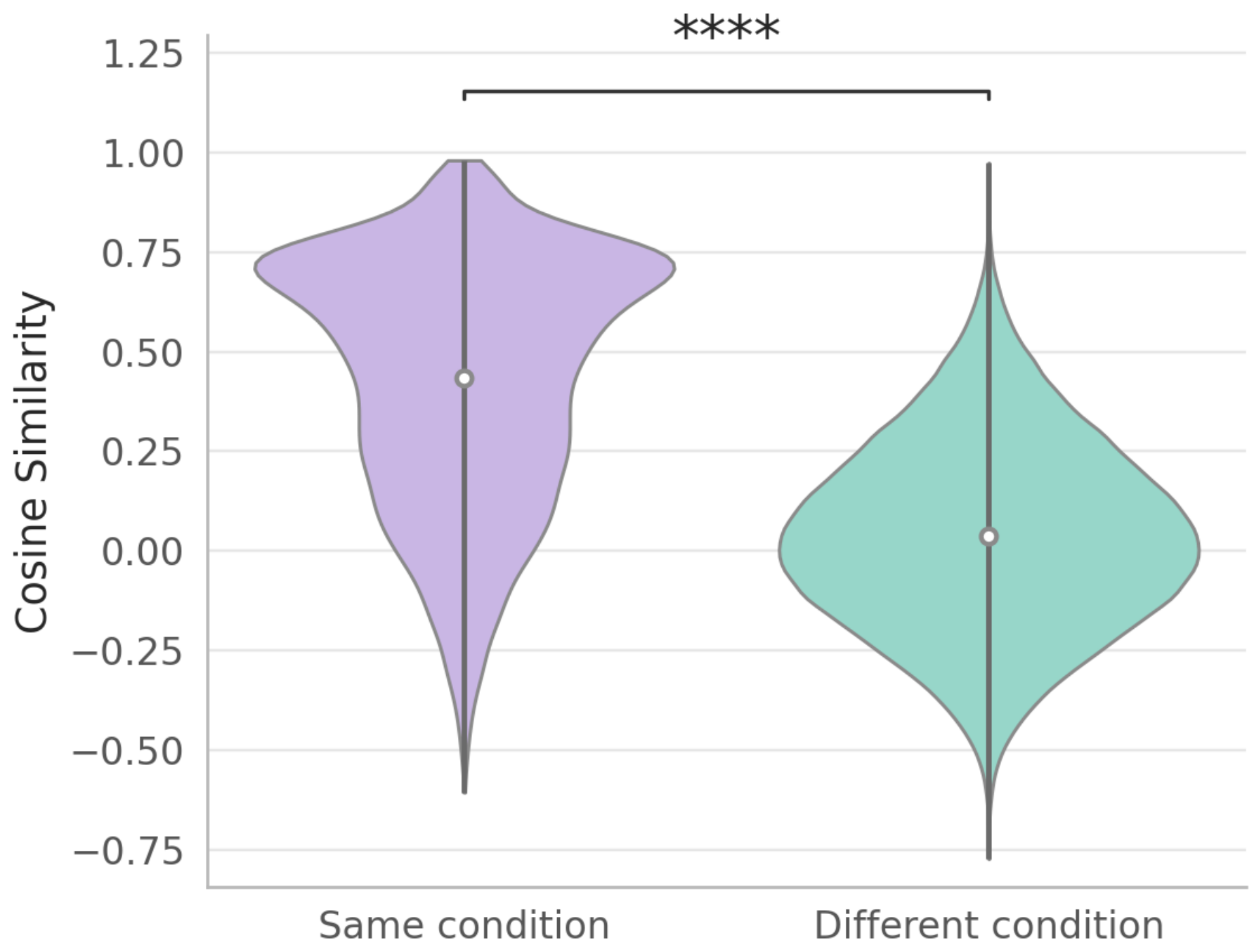

Figure S4 Pairwise cosine similarity distributions of DEG latent vectors generated from compound-induced DEG profiles across all test-set samples. The "Same condition" distribution represents similarities among latent vectors corresponding to the same compound regardless of cell line, while the "Different condition" distribution represents pairwise similarities between latent vectors from distinct compounds. A Mann-Whitney U test confirmed a statistically significant difference between the two distributions (****$p < 0.0001$).

S8. Interpretation of DEG encoder

To investigate whether the DEG encoder captures biologically distinct perturbation signatures, we examined the latent vectors and layer-level activation patterns of the DEG AE using held-out test samples from the A549 cell line. Compounds were first grouped according to their mechanisms of action, and two pharmacological groups were selected for representative analysis: proteasome inhibitors (MG-132 and bortezomib) and receptor- and second-messenger modulating compounds (H-89, forskolin, telmisartan, valsartan, and anagrelide). Visualization of the 64-dimensional latent vectors showed that samples from the two groups were largely organized into distinct clusters (Fig. S5A).

To assess whether this separation was also reflected in biologically interpretable features, we examined activation values of GO term-aligned nodes in the first encoder layer. The top 20 discriminative GO terms per group were selected, and their sample-level activation profiles were visualized using hierarchical clustering and heatmaps (Fig. S5B), with a complete list provided in Table S5.

Among the top-discriminative GO terms for proteasome inhibitor-treated samples, several were consistent with stress-adaptive and protein quality control processes, including proteolysis involved in protein catabolic process (GO:0051603), regulation of response to endoplasmic reticulum stress (GO:1905897), regulation of cellular response to heat (GO:1900034), regulation of amino acid transport (GO:0051955), and autophagosome maturation (GO:0097352), consistent with the established role of proteasome inhibition in disrupting the ubiquitin-proteasome system, inducing proteostasis imbalance, and engaging compensatory stress-response pathways [3,4,5,6 ,7 ,8 ,9 ,10 ,11]. Disruption of Golgi membrane dynamics was also reflected among the top-ranked terms, including intra-Golgi vesicle-mediated transport (GO:0006891) and Golgi inheritance (GO:0048313), consistent with evidence linking proteasomal regulation to Golgi organization and stress-induced Golgi dispersal [12]. Additionally, positive regulation of mitotic cell cycle (GO:0045931) appeared among the top-discriminative terms, likely reflecting impaired cyclin turnover and consequent cell-cycle dysregulation [13].

In contrast, among the top-discriminative GO terms for receptor- and second-messenger modulating compounds, several were associated with extracellular signaling and membrane-regulatory processes. These included cellular response to prostaglandin E stimulus (GO:0071380), neuropeptide signaling pathway (GO:0007218), regulation of transmission of nerve impulse (GO:0051969), cholesterol transport (GO:0030301), and monoatomic cation transmembrane transport (GO:0098655). These functional categories are consistent with receptor-coupled and cAMP-PKA-mediated signaling processes that influence membrane-regulatory functions and ion flux [14,15,16,17,18,19]. Immune- and stress-related terms, including negative regulation of dendritic cell antigen processing and presentation (GO:0002605) and negative regulation of defense response (GO:0031348), were also represented among the top-ranked terms, suggesting broader downstream inflammatory or stress-responsive transcriptional correlates rather than direct cell type-specific programs [20].

Together, these results indicate that the DEG encoder organizes its latent space in a pharmacologically meaningful way, with group-specific activation patterns aligning with known mechanisms of action. This biological structure in the latent vector suggests a potential application for generating functional hypotheses about the perturbation targets of generated molecules, complementing structural similarity-based evaluation with a pathway-level perspective. However, these activation patterns should be interpreted with caution: elevated activation of a GO term-aligned node reflects co-variation with pharmacological class in transcriptional space and may include indirect downstream effects or compensatory cellular responses, rather than constituting direct evidence of pathway activation or mechanistic drug action.

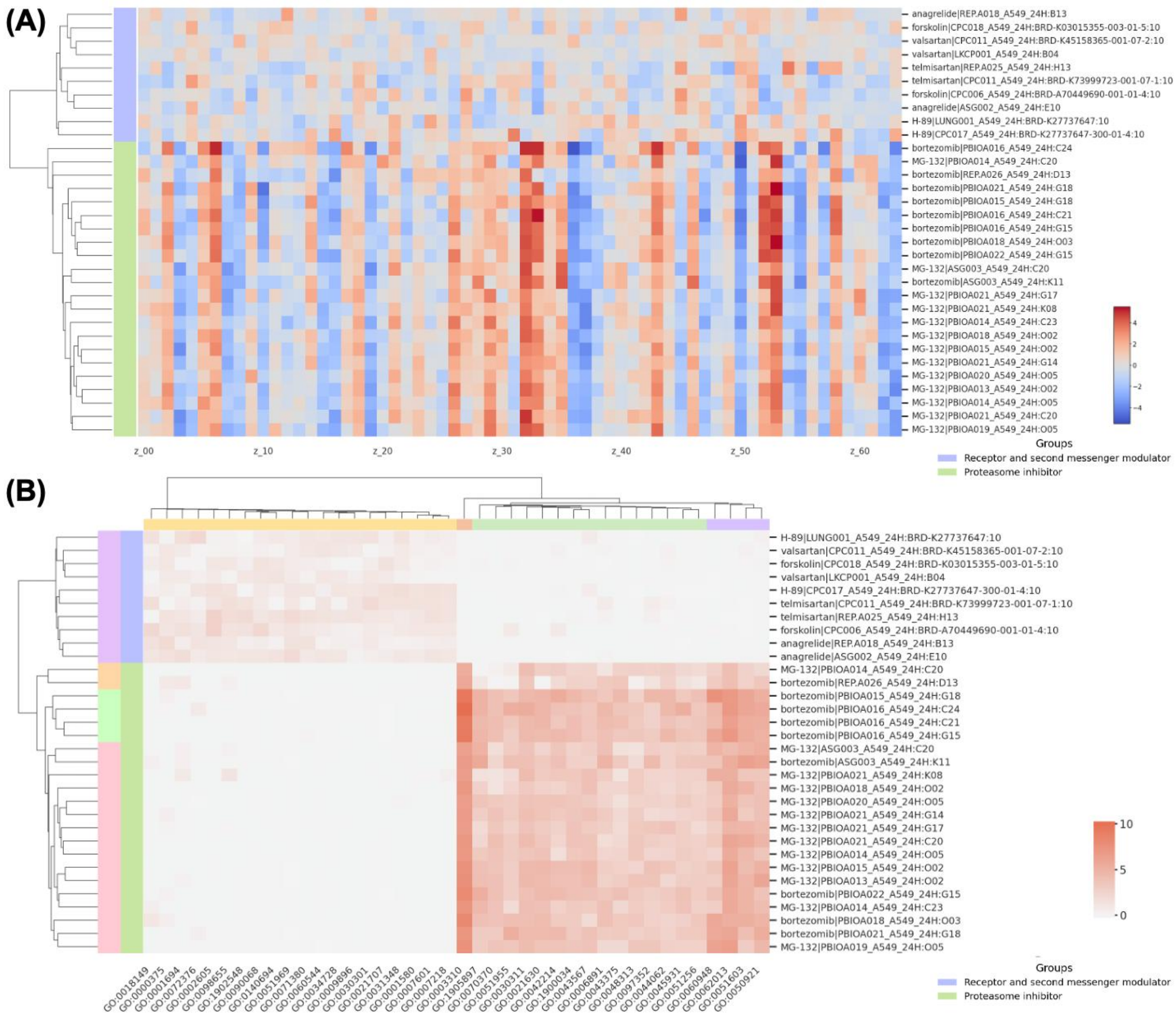

Figure S5 Sample-level hierarchical clustering of group-discriminative latent and GO term-aligned nodes. (A) Heatmap and hierarchical clustering of sample-level 64-dimensional latent vectors from the DEG encoder for two predefined perturbation groups. Samples were clustered across all latent dimensions without condition averaging; row annotations indicate group identity. (B) Heatmap and hierarchical clustering of sample-level layer 1 activation values for the top 20 GO terms per group that most strongly distinguished the two perturbation groups. Samples were clustered across the combined set of discriminative GO terms; row annotations indicate group identity and column annotations indicate GO-term clusters.

Table S5 Top 20 group-specific GO terms selected based on between-group activation differences in the DEG encoder.

Enriched group	GO term	GO name	Rank within group
Receptor- and second-messenger modulating compounds	GO:0002605	negative regulation of dendritic cell antigen processing and presentation	1
	GO:0051969	regulation of transmission of nerve impulse	2
	GO:0090068	positive regulation of cell cycle process	3
	GO:0030301	cholesterol transport	4
	GO:0071380	cellular response to prostaglandin E stimulus	5
	GO:0031348	negative regulation of defense response	6
	GO:0007601	visual perception	7
	GO:0098655	monoatomic cation transmembrane transport	8
	GO:0060544	regulation of necroptotic process	9
	GO:0140694	membraneless organelle assembly	10
	GO:0021707	cerebellar granule cell differentiation	11
	GO:0018149	peptide cross-linking	12
	GO:0007218	neuropeptide signaling pathway	13

	GO:0000375	RNA splicing, via transesterification reactions	14
	GO:1902548	negative regulation of cellular response to vascular endothelial growth factor stimulus	15
	GO:0034728	nucleosome organization	16
	GO:0072376	protein activation cascade	17
	GO:0009896	positive regulation of catabolic process	18
	GO:0001580	detection of chemical stimulus involved in sensory perception of bitter taste	19
	GO:0001694	histamine biosynthetic process	20
**Proteasome inhibitors**	GO:0003310	pancreatic A cell differentiation	1
	GO:0062013	positive regulation of small molecule metabolic process	2
	GO:0051603	obsolete proteolysis involved in protein catabolic process	3
	GO:0050921	positive regulation of chemotaxis	4
	GO:0060948	cardiac vascular smooth muscle cell development	5
	GO:0030311	poly-N-acetyllactosamine biosynthetic process	6
	GO:0021630	olfactory nerve maturation	7
	GO:1905897	regulation of response to endoplasmic reticulum stress	8
	GO:1900034	regulation of cellular response to heat	9
	GO:0043567	regulation of insulin-like growth factor receptor signaling pathway	10
	GO:0042214	terpene metabolic process	11
	GO:0070370	cellular heat acclimation	12
	GO:0045931	positive regulation of mitotic cell cycle	13
	GO:0006891	intra-Golgi vesicle-mediated transport	14
	GO:0048313	Golgi inheritance	15
	GO:0051955	regulation of amino acid transport	16
	GO:0097352	autophagosome maturation	17
	GO:0051256	mitotic spindle midzone assembly	18
	GO:0043375	CD8-positive, alpha-beta T cell lineage commitment	19
	GO:0044062	regulation of excretion	20

S9. Ablation Study

We performed an ablation study under the scaffold split to quantify the contributions of the GO-BP hierarchy and DEG conditioning to DEG2MOL (Table S6). Removing the GO-BP hierarchical structure from the DEG encoder (w/o GO) increased uniqueness from 0.8076 ± 0.0195 to 0.9915 ± 0.0078 and scaffold diversity from 0.5710 ± 0.0362 to 0.6871 ± 0.0069, while novelty increased slightly from 0.9992 ± 0.0001 to 1.0000 ± 0.0000. However, molecular similarity decreased consistently across ECFP6 (0.2214 ± 0.0039 to 0.2052 ± 0.0037), MACCS (0.6806 ± 0.0030 to 0.6761 ± 0.0029), and Fraggle (0.6291 ± 0.0017 to 0.6244 ± 0.0068). Internal diversity also decreased slightly from 0.8772 ± 0.0026 to 0.8730 ± 0.0038. These results suggest that the GO-BP hierarchy acts as a biologically informed constraint that directs the learned representation toward a more focused chemical space with greater structural similarity to the reference molecules, although this constraint reduces uniqueness and scaffold-level diversity.

We next conducted a negative-control experiment in which the input DEG values were randomly shuffled feature-wise. DEG shuffling produced a larger decline in molecular similarity, reducing ECFP6 from 0.2214 ± 0.0039 to 0.1860 ± 0.0006, MACCS from 0.6806 ± 0.0030 to 0.6466 ± 0.0022, and Fraggle from 0.6291 ± 0.0017 to 0.6096 ± 0.0019. In contrast, uniqueness increased from 0.8076 ± 0.0195 to 0.9794 ± 0.0034, internal diversity from 0.8772 ± 0.0026 to 0.8812 ± 0.0011, and scaffold diversity from 0.5710 ± 0.0362 to 0.7476 ± 0.0122. Novelty also increased slightly from 0.9992 ± 0.0001 to 0.9997 ± 0.0000. The consistent loss of similarity after disrupting the correspondence between DEG features and experimental conditions indicates that DEG2MOL uses condition-specific transcriptional information to guide molecular generation rather than relying solely on a generic molecular distribution. Overall, the complete DEG2MOL model achieved the highest ECFP6, MACCS, and Fraggle similarities, whereas both ablations increased uniqueness and scaffold diversity. These results support complementary roles for the GO-BP hierarchy and DEG conditioning in directing molecular generation toward reference-aligned chemical space.

Table S6 Ablation study. Cascaded metrics (%Val, %V+U, %V+U+N) are computed by sequentially multiplying validity, uniqueness, and novelty. ECFP6, MACCS, and Fraggle denote structural similarity scores, where the maximum similarity among the generated molecules is computed for each reference compound and then averaged across all reference compounds. Abbreviations: Val, Validity; Uniq, Uniqueness; Novel, Novelty; IntDiv, Internal Diversity; ScafDiv, Scaffold Diversity.

Model	%Val	%Uniq	%Nov	%V+U	%V+U+N	IntDiv	ScafDiv	ECFP6	MACCS	Fraggle
**DEG2MOL**	<u>0.9964 ± 0.0009</u>	0.8076 ± 0.0195	0.9992 ± 0.0001	0.8047 ± 0.0194	0.8040 ± 0.0194	<u>0.8772 ± 0.0026</u>	0.5710 ± 0.0362	**0.2214 ± 0.0039**	**0.6806 ± 0.0030**	**0.6291 ± 0.0017**
**w/o GO**	**0.9969 ± 0.0005**	**0.9915 ± 0.0078**	**1.0000 ± 0.0000**	**0.9884 ± 0.0078**	**0.9884 ± 0.0078**	0.8730 ± 0.0038	<u>0.6871 ± 0.0069</u>	<u>0.2052 ± 0.0037</u>	<u>0.6761 ± 0.0029</u>	<u>0.6244 ± 0.0068</u>
**DEG shuffling**	0.9951 ± 0.0003	<u>0.9794 ± 0.0034</u>	<u>0.9997 ± 0.0000</u>	<u>0.9746 ± 0.0034</u>	<u>0.9743 ± 0.0034</u>	**0.8812 ± 0.0011**	**0.7476 ± 0.0122**	0.1860 ± 0.0006	0.6466 ± 0.0022	0.6096 ± 0.0019

References

[1] Aleksander, S. A. et al. The gene ontology knowledgebase in 2023. Genetics, 224(1):iyad031, 2023.

[2] Luecken MD, Büttner M, Chaichoompu K, Danese A, Interlandi M, Müller MF, Strobl DC, Zappia L, Dugas M, Colomé-Tatché M, Theis FJ. Benchmarking atlas-level data integration in single-cell genomics. Nature methods. 2022 Jan;19(1):41-50.

[3] Aliabadi, F. et al. Ubiquitin–proteasome system and the role of its inhibitors in cancer therapy. Open Biology, 11(4), 2021.

[4] Ding, W.-X. et al. Linking of autophagy to ubiquitin-proteasome system is important for the regulation of endoplasmic reticulum stress and cell viability. The American journal of pathology, 171(2):513–524, 2007.

[5] Glickman, M. H. & Ciechanover, A. The ubiquitin-proteasome proteolytic pathway: destruction for the sake of construction. Physiological reviews, 2002.

[6] Hershko, A. & Ciechanover, A. The ubiquitin system. Annual review of biochemistry, 67(1):425–479, 1998.

[7] Kim, H.-J. et al. Systemic analysis of heat shock response induced by heat shock and a proteasome inhibitor mg132. PloS one, 6(6):e20252, 2011.

[8] Labbadia, J. & Morimoto, R. I. The biology of proteostasis in aging and disease. Annual review of biochemistry, 84(1):435–464, 2015.

[9] Qu, J. et al. The roles of the ubiquitin–proteasome system in the endoplasmic reticulum stress pathway. International journal of molecular sciences, 22(4):1526, 2021.

[10] Richter, K. et al. The heat shock response: life on the verge of death. Molecular cell, 40(2):253–266, 2010.

[11] Suraweera, A. et al. Failure of amino acid homeostasis causes cell death following proteasome inhibition. Molecular cell, 48(2):242–253, 2012.

[12] Eisenberg-Lerner, A. et al. Golgi organization is regulated by proteasomal degradation. Nature Communications, 11:409, 2020.

[13] Bassermann, F., Eichner, R. & Pagano, M. The ubiquitin proteasome system – implications for cell cycle control and the targeted treatment of cancer. Cell Cycle, 13(22):3521–3532, 2014.

[14] Betke, K. M. et al. Gpcr mediated regulation of synaptic transmission. Progress in neurobiology, 96(3):304–321, 2012.

[15] Cheng, L. et al. Structure, function and drug discovery of gpcr signaling. mol biomed 4: 46, 2023.

[16] Hoyer, D. & Bartfai, T. Neuropeptides and neuropeptide receptors: drug targets, and peptide and non-peptide ligands: a tribute to prof. dieter seebach. Chemistry & biodiversity, 9(11):2367–2387, 2012.

[17] Narumiya, S. et al. Prostanoid receptors: structures, properties, and functions. Physiological reviews, 79(4):1193–1226, 1999.

[18] Sassone-Corsi, P. The cyclic AMP pathway. Cold Spring Harbor Perspectives in Biology, 4(12):a011148, 2012.

[19] Wright, P. T., Schobesberger, S., Tzanopoulou, E. & Gorelik, J. Studying GPCR/cAMP pharmacology from the perspective of cellular structure. Frontiers in Pharmacology, 6:148, 2015.

[20] Saavedra, J. M. Angiotensin receptor blockers are not just for hypertension anymore. Physiology (Bethesda), 36(3):160–173, 2021.
